# Supplementary material for: Theoretical framework for confined ion transport in two-dimensional nanochannels
Source: Nat Commun. 2025 Jul 20;16:6675. doi: 10.1038/s41467-025-61735-9 (PMC12276316; doi:10.1038/s41467-025-61735-9)
Supplement: Supplementary file 1 — Supplementary Information [file 41467_2025_61735_MOESM1_ESM.pdf]

SUPPLEMENTARY INFORMATION

Theoretical Framework for Confined Ion Transport in Two-dimensional Nanochannels

Shouwei Liao[1], Yanchang Liu[1], Libo Li[1]*, Li Ding[2], Yanying Wei[1,3]* & Haihui Wang[2]*

[1] State Key Laboratory of Pulp and Paper Engineering, School of Chemistry & Chemical Engineering, Guangdong Provincial Key Lab of Green Chemical Product Technology, South China University of Technology, Guangzhou 510610, China.

[2] Beijing Key Laboratory of Membrane Materials and Engineering, Department of Chemical Engineering, Tsinghua University, Beijing 100084, China.

[3] Quzhou Membrane Material Innovation Institute, Quzhou 324000, China

*e-mail: celbli@scut.edu.cn; ceyywei@scut.edu.cn; cehhwang@tsinghua.edu.cn

Table of contents

Supplementary Table 1 3
Supplementary Fig. 1 4
Supplementary Table 2 5
Supplementary Table 3 6
Supplementary Fig. 2 7
Supplementary Fig. 3 8
Supplementary Fig. 4 9
Supplementary Fig. 5 10
Supplementary Fig. 6 11
Supplementary Fig. 7 12
Supplementary Fig. 8 13
Supplementary Fig. 9 14
Supplementary Note 1 15
Supplementary Fig. 10 16
Supplementary Note 2 17
Supplementary Fig. 11 18
Supplementary Fig. 12 19
Supplementary Fig. 13 20
Supplementary Fig. 14 21
Supplementary Fig. 15 22
Supplementary Fig. 16 23
Supplementary Fig. 17 24
Supplementary Fig. 18 25
Supplementary Table 4 26
Supplementary Table 5 27
Supplementary Table 6 28
Supplementary Table 7 29
Supplementary Note 3 30
Supplementary Note 4 31
Supplementary Fig. 19 33
Supplementary Fig. 20 34
Supplementary Fig. 21 35
Supplementary Note 5 36
Supplementary Fig. 22 37
Supplementary Fig. 23 38
Supplementary Fig. 24 39
Supplementary Fig. 25 40
Supplementary Table 8 41
Supplementary Fig. 26 43
Supplementary Fig. 27 44
Supplementary References 45

Supplementary Table 1 | The Lennard-Jones (LJ) parameters and charges for each atom type in molecular dynamics simulations.

Atom type [a]		σ (nm)	ε (kJ/mol)	Charge (e)
Merz FF[1,2]	Li^+	0.234	0.025	+1
	Na^+	0.261	0.122	+1
	K^+	0.311	0.712	+1
	Rb^+	0.324	0.961	+1
	Cs^+	0.356	1.629	+1
	Ca^{2+}	0.286	0.349	+2
	Mg^{2+}	0.249	0.062	+2
	Cl^-	0.385	2.224	-1
Netz FF[3,4]	Li^+	0.287	0.000615	+1
	Na^+	0.381	0.000615	+1
	K^+	0.453	0.000615	+1
	Rb^+	-	-	+1
	Cs^+	0.517	0.000615	+1
	Ca^{2+}	0.241	0.935	+2
	Mg^{2+}	0.163	0.591	+2
	Cl^-	0.439	0.416	-1
Williams FF[5]	Li^+	0.141	1.409	+1
	Na^+	0.216	1.475	+1
	K^+	0.284	1.798	+1
	Rb^+	-	-	+1
	Cs^+	-	-	+1
	Ca^{2+}	0.241	0.94	+2
	Mg^{2+}	0.163	0.59	+2
	Cl^-	0.493	0.054	-1
OPLS-AA[6]	Li^+	0.213	0.0765	+1
	Na^+	0.333	0.0116	+1
	K^+	0.493	0.00137	+1
	Rb^+	0.562	0.000715	+1
	Cs^+	0.672	0.000339	+1
	Ca^{2+}	0.287	0.349	+2
	Mg^{2+}	0.252	0.0624	+2
	Cl^-	0.442	0.493	-1
SPC/E water model[7]	O	0.3166	0.65	-0.8476
	H	-	-	+0.4238
Graphene[8]	C	0.3214	0.49	0
hBN[9]	B	0.33087	0.2897	+0.907
	N	0.32174	0.1979	-0.907
MoS_2[10]	Mo	0.42	0.0565	+0.6
	S	0.313	1.93	-0.3
$g\text{-}C_3N_4$[11]	C	0.343	0.44	+0.1916
	N1 [b]	0.326	0.289	-0.1883
	N2 [b]	0.326	0.289	-0.1373
	N3 [b]			

[a]: Two different LJ parameters between ion and graphene, $\varepsilon_{\mathrm{I-W}}^{\mathrm{ion-\pi}}$ and $\varepsilon_{\mathrm{I-W}}^{\mathrm{LB}}$, were employed (see Supplementary Table 2). If not otherwise stated, the LJ parameters between different atoms were derived from LB mixing rule. The interaction energy (electrostatic interaction + LJ interaction) between ions and wall atoms are calculated as: $U_{I-W}(r_{\mathrm{I-W}}) = \frac{q_{\mathrm{ion}}q_{\mathrm{wall}}}{4\pi\epsilon r_{\mathrm{I-W}}} + 4\varepsilon_{\mathrm{I-W}}[\left(\frac{\sigma_{\mathrm{I-W}}}{r_{\mathrm{I-W}}}\right)^{12} - \left(\frac{\sigma_{\mathrm{I-W}}}{r_{\mathrm{I-W}}}\right)^{6}]$, where $r_{\mathrm{I-W}}$ is the distance between ions and wall atoms; q_{ion} and q_{ion} are charges of ion and wall atom, respectively; ϵ is vacuum permittivity; $\varepsilon_{\mathrm{I-W}}$ and $\sigma_{\mathrm{I-W}}$ are LJ parameters between ions and wall atoms.

[b]: See Supplementary Fig. 6 to distinguish N1, N2, N3 atoms of $g\text{-}C_3N_4$.

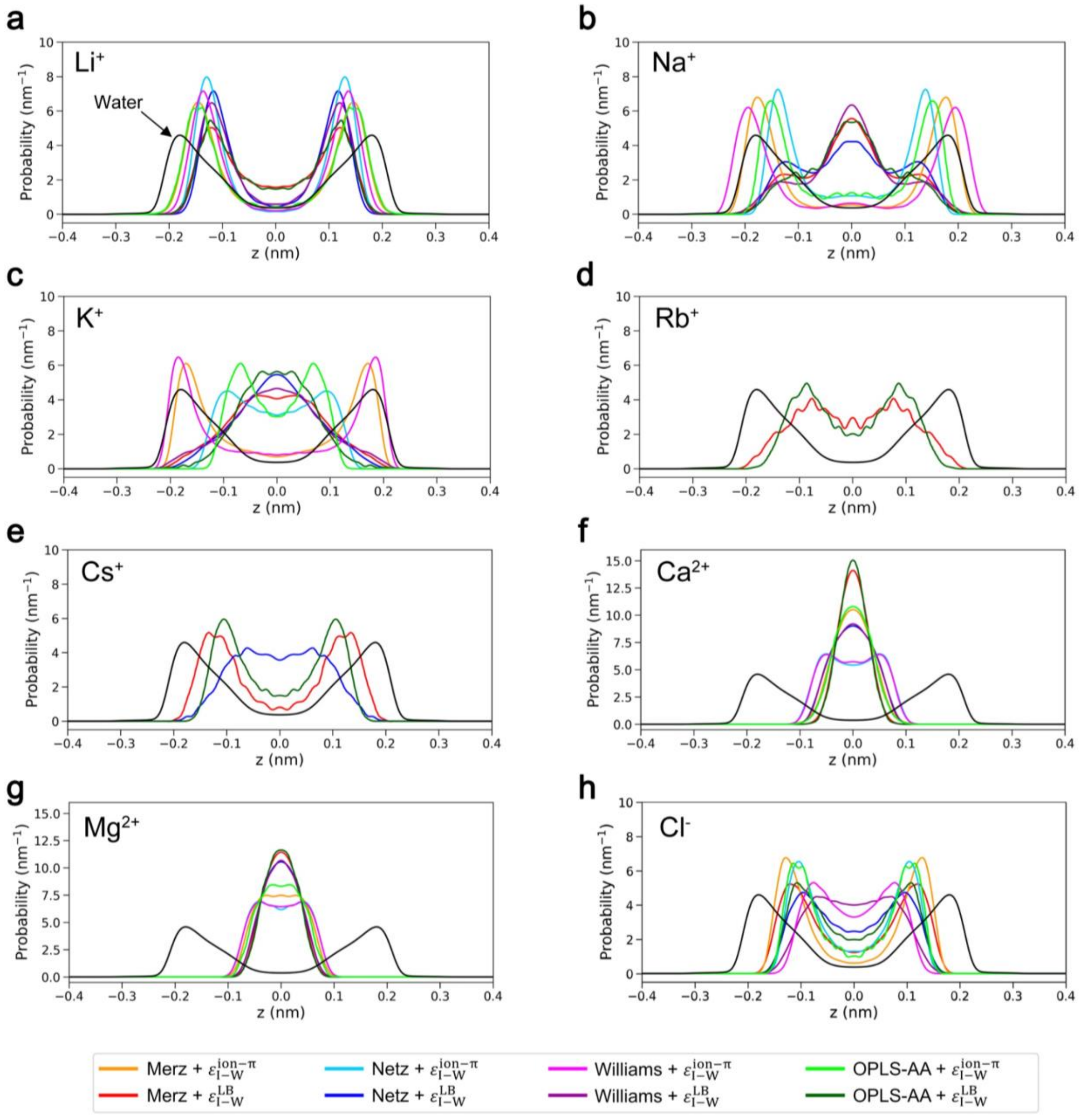

Supplementary Fig. 1 | Distribution profiles of (a) Li^+, (b) Na^+, (c) K^+, (d) Rb^+, (e) Cs^+, (f) Ca^{2+}, (g) Mg^{2+} or (h) Cl^- in graphene 2D nanochannel simulated with Merz FF, Netz FF, Williams FF or OPLS-AA FF. For each FF, two different LJ parameters between ion and graphene, $\varepsilon_{I-W}^{ion-\pi}$ and ε_{I-W}^{LB} (see Supplementary Table 2), were employed. The black curves are distribution profiles of oxygen atoms of water molecules. Source data are provided as a Source Data file.

Supplementary Table 2 | Ion-graphene Lennard-Jones (LJ) interaction parameters of studied ions simulated with $\varepsilon_{\mathrm{I-W}}^{\mathrm{ion-\pi}}$[b] or $\varepsilon_{\mathrm{I-W}}^{\mathrm{LB}}$[b] parameters.

Force field[a]	Ion	$\sigma_{\mathrm{I-W}}$ [b] (nm)	$\varepsilon_{\mathrm{I-W}}^{\mathrm{ion-\pi}}$[b] (KJ/mol)	$\varepsilon_{\mathrm{I-W}}^{\mathrm{LB}}$[b] (KJ/mol)	r_{HS} [c] (nm)
Merz[1,2]	Li^+	0.2805	1.5000	0.1104	0.208
	Na^+	0.2924	1.9500	0.2442	0.234
	K^+	0.3161	1.8300	0.5906	0.276
	Rb^+	0.3179	-	0.7480	0.290
	Cs^+	0.3323	-	1.1205	0.312
	Ca^{2+}	0.3043	4.1000	0.4134	0.244
	Mg^{2+}	0.2868	5.5000	0.1748	0.208
	Cl^-	0.3552	1.4800	1.0438	0.320
Netz[3,4]	Li^+	0.3042	0.8390	0.0173	0.192
	Na^+	0.3512	0.9254	0.0173	0.228
	K^+	0.3872	0.8259	0.0173	0.254
	Cs^+	0.4195	-	0.0173	0.280
	Ca^{2+}	0.2812	5.2420	0.6786	0.232
	Mg^{2+}	0.2422	14.4476	0.5376	0.196
	Cl^-	0.3807	1.0398	0.4482	0.322
Williams[5]	Li^+	0.2312	4.0000	0.8310	0.198
	Na^+	0.2687	3.0000	0.8500	0.238
	K^+	0.3027	2.2000	0.9386	0.274
	Ca^{2+}	0.2812	5.0000	0.6786	0.232
	Mg^{2+}	0.2422	14.2500	0.5376	0.196
	Cl^-	0.4022	0.6000	0.1627	0.314
OPLS-AA[6]	Li^+	0.2670	1.9939	0.1936	0.208
	Na^+	0.3272	1.2227	0.0754	0.240
	K^+	0.4074	0.8177	0.0259	0.280
	Rb^+	0.4418	-	0.0187	0.304
	Cs^+	0.4965	-	0.0129	0.336
	Ca^{2+}	0.3043	4.1950	0.1749	0.244
	Mg^{2+}	0.2868	4.9133	0.4134	0.208
	Cl^-	0.3552	0.9820	0.5661	0.328

[a]: The Lennard-Jones (LJ) parameters are taken from the literatures. Parameters for some ions may be missing in some FFs, *e.g.* Rb^+'s parameters are missing in Merz FF.

[b]: $\sigma_{\mathrm{I-W}}$ is the LJ parameter σ between ion and graphene wall atoms, derived from Lorentz-Berthelot (LB) mixing rule: $\sigma_{\mathrm{I-W}} = (\sigma_{\mathrm{ion}} + \sigma_{\mathrm{wall}})/2$ where σ_{ion} and σ_{wall} are the σ parameters of ion and wall atoms, respectively (see Supplementary Table 1). Two versions of LJ parameters ε between ions and graphene were employed: the original version derived from LB mixing rule, denoted as $\varepsilon_{\mathrm{I-W}}^{\mathrm{LB}}$ ($\varepsilon_{\mathrm{I-W}}^{\mathrm{LB}} = \sqrt{\varepsilon_{\mathrm{ion}} \cdot \varepsilon_{\mathrm{wall}}}$ where $\varepsilon_{\mathrm{ion}}$ and $\varepsilon_{\mathrm{wall}}$ are ε parameters of ion and wall atoms, respectively, as shown in Supplementary Table 1); another optimized version taken from refs.[5,12], denoted as $\varepsilon_{\mathrm{I-W}}^{\mathrm{ion-\pi}}$. $\varepsilon_{\mathrm{I-W}}^{\mathrm{ion-\pi}}$ parameters of OPLS-AA are calculated with the optimization method in ref. [5]. The interaction energy between atoms of different molecules/ions are calculated similarly to the footnote [a] of Supplementary Table 1.

[c]: r_{HS} is the radius of the 1st hydration shell, denoted as the position of the 1st valley of corresponding RDF curve in Supplementary Figs. 2-5.

Supplementary Table 3 | Diffusivity of ions in graphene 2D nanochannel (D_{channel}) or bulk water (D_{bulk}) simulated in this work.

Force field	Ion	$D_{\mathrm{channel}}^{\mathrm{LB}}$ [a] (10^{-9} m^2/s)	$D_{\mathrm{channel}}^{\mathrm{ion-\pi}}$ [a] (10^{-9} m^2/s)	D_{bulk} (10^{-9} m^2/s)	$\frac{D_{\mathrm{channel}}^{\mathrm{LB}}}{D_{\mathrm{bulk}}}$	$\frac{D_{\mathrm{channel}}^{\mathrm{ion-\pi}}}{D_{\mathrm{bulk}}}$
Merz[1,2]	Li^+	1.045 ± 0.069	0.988 ± 0.077	1.219 ± 0.147	0.857	0.811
	Na^+	1.071 ± 0.059	0.866 ± 0.113	1.058 ± 0.046	1.012	0.819
	K^+	1.393 ± 0.101	1.445 ± 0.174	1.856 ± 0.107	0.751	0.779
	Rb^+	1.295 ± 0.058	-	1.409 ± 0.119	0.919	-
	Cs^+	1.129 ± 0.044	-	1.579 ± 0.110	0.715	-
	Ca^{2+}	0.612 ± 0.024	0.637 ± 0.086	0.602 ± 0.031	1.017	1.058
	Mg^{2+}	0.597 ± 0.042	0.631 ± 0.056	0.569 ± 0.054	1.049	1.109
	Cl^-	1.392 ± 0.097	1.290 ± 0.094	1.479 ± 0.109	0.941	0.872
Netz[3,4]	Li^+	0.791 ± 0.119	0.844 ± 0.041	0.939 ± 0.058	0.842	0.899
	Na^+	1.034 ± 0.057	0.964 ± 0.076	1.053 ± 0.043	0.982	0.915
	K^+	1.279 ± 0.084	1.367 ± 0.138	1.503 ± 0.042	0.851	0.91
	Cs^+	1.410 ± 0.080	-	1.548 ± 0.164	0.911	-
	Ca^{2+}	0.686 ± 0.055	0.707 ± 0.047	0.669 ± 0.036	1.025	1.057
	Mg^{2+}	0.590 ± 0.065	0.613 ± 0.054	0.566 ± 0.024	1.042	1.083
	Cl^-	1.331 ± 0.087	1.311 ± 0.073	1.538 ± 0.089	0.865	0.852
Williams[5]	Li^+	0.924 ± 0.081	0.889 ± 0.038	1.135 ± 0.074	0.814	0.783
	Na^+	1.048 ± 0.120	0.886 ± 0.069	1.083 ± 0.086	0.968	0.818
	K^+	1.308 ± 0.039	1.305 ± 0.086	1.652 ± 0.116	0.792	0.79
	Ca^{2+}	0.659 ± 0.050	0.650 ± 0.069	0.647 ± 0.048	1.019	1.005
	Mg^{2+}	0.627 ± 0.028	0.615 ± 0.061	0.588 ± 0.042	1.066	1.046
	Cl^-	1.333 ± 0.089	1.393 ± 0.163	1.404 ± 0.053	0.949	0.992
OPLS-AA[6]	Li^+	0.952 ± 0.036	0.867 ± 0.071	1.101 ± 0.134	0.865	0.787
	Na^+	1.088 ± 0.058	0.873 ± 0.099	1.043 ± 0.064	1.043	0.837
	K^+	1.426 ± 0.104	1.470 ± 0.201	1.778 ± 0.127	0.802	0.827
	Rb^+	1.394 ± 0.197	-	1.849 ± 0.092	0.754	-
	Cs^+	1.154 ± 0.117	-	1.526 ± 0.082	0.756	-
	Ca^{2+}	0.663 ± 0.035	0.629 ± 0.109	0.636 ± 0.130	1.042	0.989
	Mg^{2+}	0.579 ± 0.039	0.583 ± 0.069	0.591 ± 0.063	0.98	0.986
	Cl^-	1.286 ± 0.084	1.318 ± 0.106	1.497 ± 0.134	0.859	0.88

[a]: $D_{\mathrm{channel}}^{\mathrm{LB}}$ and $D_{\mathrm{channel}}^{\mathrm{ion-\pi}}$ stand for D_{channel} when simulated with $\varepsilon_{\mathrm{I-W}}^{\mathrm{LB}}$ and $\varepsilon_{\mathrm{I-W}}^{\mathrm{ion-\pi}}$ parameters (shown in Supplementary Table 2), respectively.

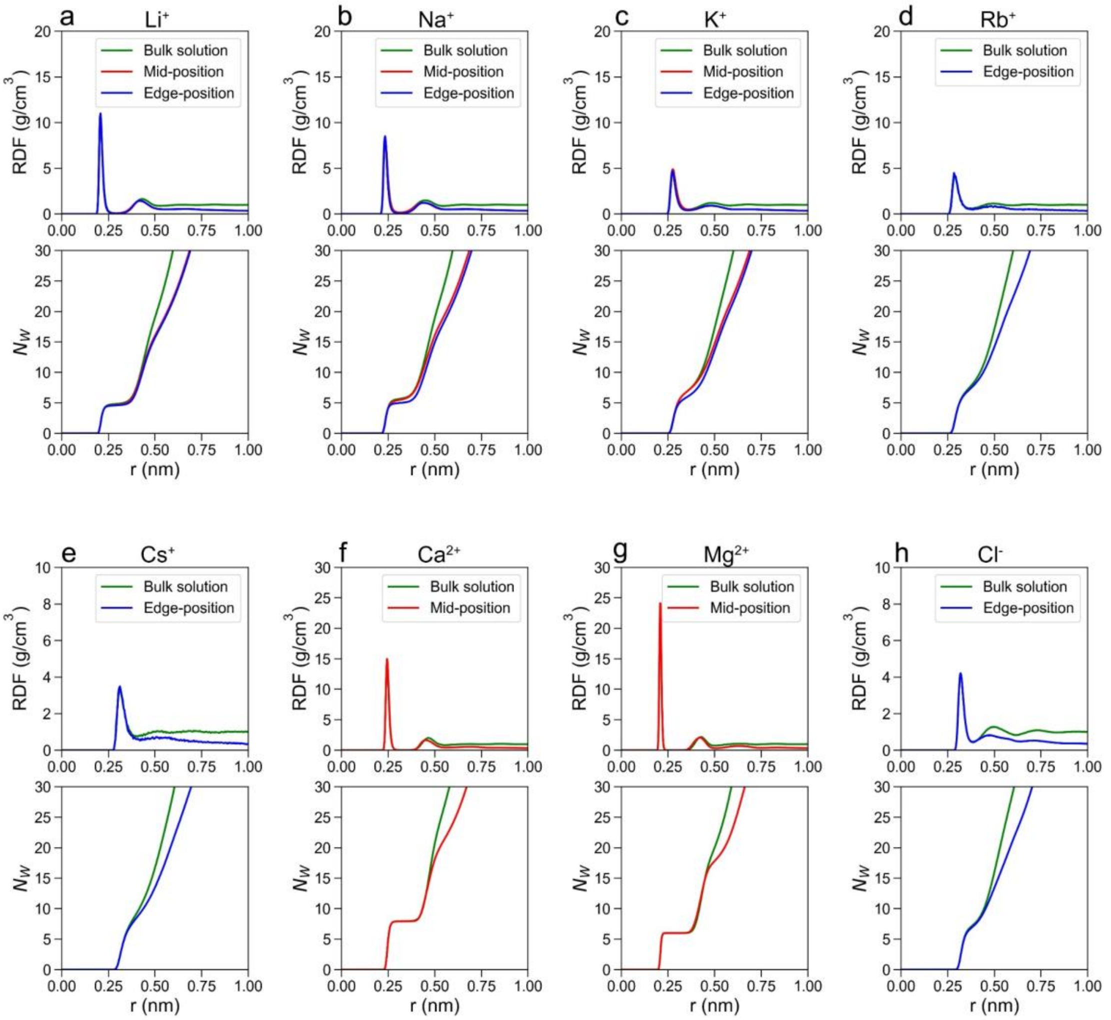

Supplementary Fig. 2 | Radial distribution function (RDF) of ion-(water) oxygen atoms in bulk solution (green) or graphene nanochannels (red: mid-position; blue: edge-position) for (a) Li^+, (b) Na^+, (c) K^+, (d) Rb^+, (e) Cs^+, (f) Ca^{2+}, (g) Mg^{2+} or (h) Cl^- and corresponding water coordination numbers (N_W) versus radial distance (r) from the ion. Simulations were performed with Merz force field. For a given ion, its r_{HS} corresponds to the position of RDF's 1st peak, also shown in Supplementary Table 2; N_W for its 1st HS is the N_W at the position of RDF's 1st valley. Rb^+, Cs^+, and Cl^- ions locate at edge-position in graphene 2D nanochannel no matter which FF parameters are used, and thus only RDFs of edge-position are shown; similarly, for Ca^{2+} and Mg^{2+} in nanochannel, only RDFs of mid-position are shown. Note N_W for an ion's 1st HS is usually similar to the bulk solution value, which only drops by ~1 sometimes when an ion locates at the edge-position. This could be attributed to that, the space in the directions parallel to the surfaces (*e.g.* the ring part of HS in Fig. 3a) accommodates more water molecules when the ion's HS is distorted by the 2D nanochannel with tiny channel height (see latter explanations in Supplementary Note 5). Source data are provided as a Source Data file.

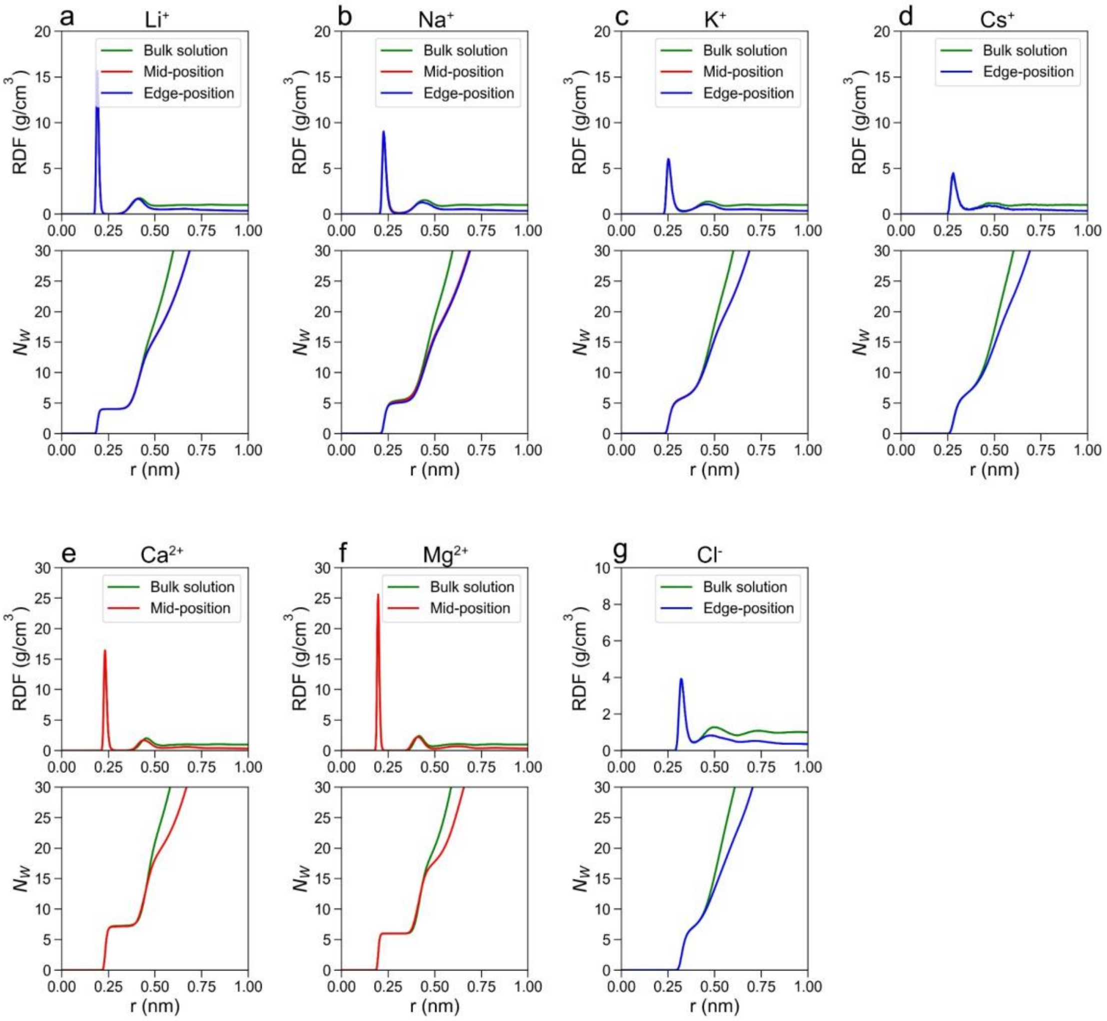

Supplementary Fig. 3 | Radial distribution function (RDF) of ion-(water) oxygen atoms in bulk solution (green) or graphene nanochannels (red: mid-position; blue: edge-position) for (a) Li^+, (b) Na^+, (c) K^+, (d) Cs^+, (e) Ca^{2+}, (f) Mg^{2+} or (g) Cl^- and corresponding water coordination numbers (N_W) versus radial distance (r) from the ion. Simulations were performed with Netz force field. For a given ion, its r_{HS} corresponds to the position of RDF's 1st peak, also shown in Supplementary Table 2; N_W for its 1st HS is the N_W at the position of RDF's 1st valley. Cs^+, and Cl^- ions locate at edge-position in graphene 2D nanochannel no matter which FF parameters are used, and thus only RDFs of edge-position are shown; similarly, for Ca^{2+} and Mg^{2+} in nanochannel, only RDFs of mid-position are shown. Source data are provided as a Source Data file.

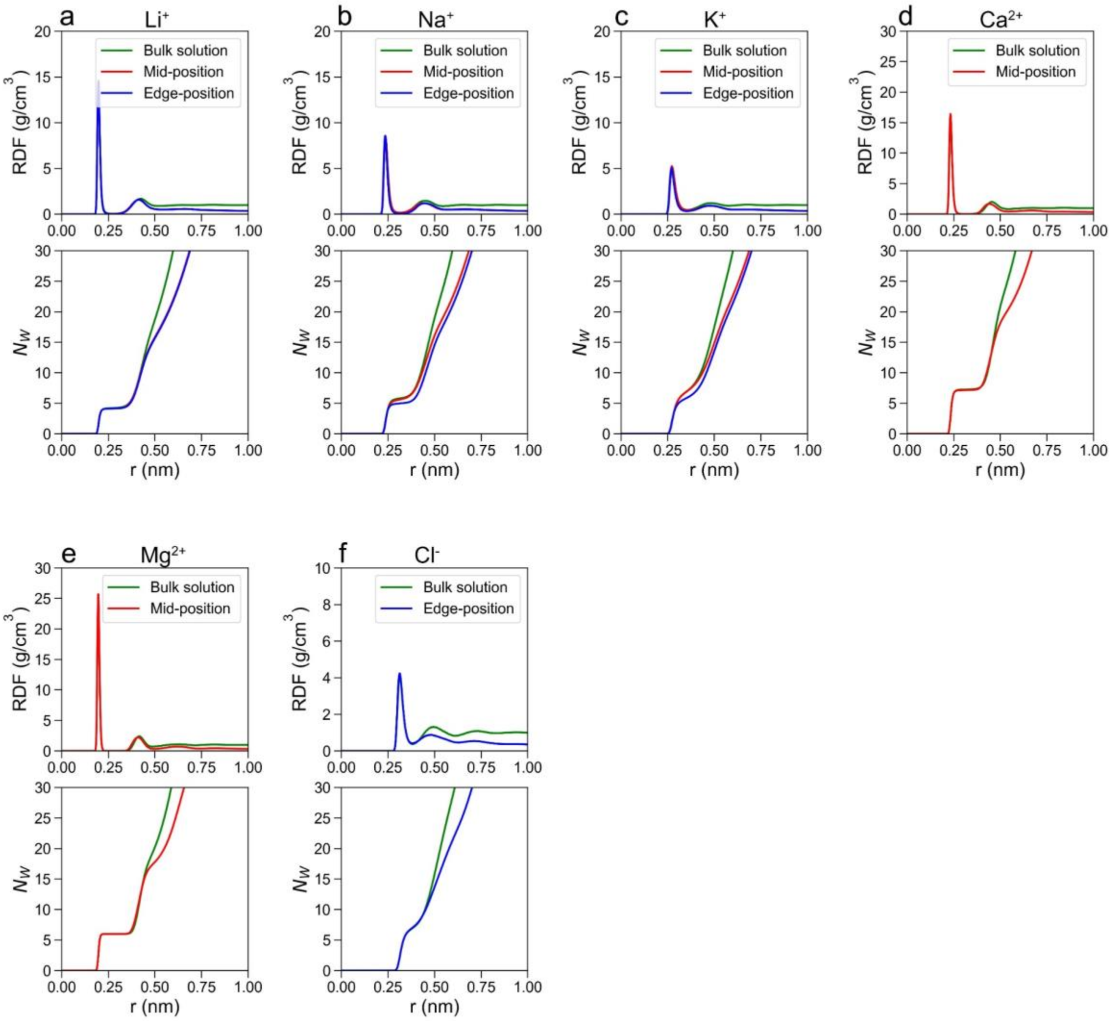

Supplementary Fig. 4 | Radial distribution function (RDF) of ion-(water) oxygen atoms in bulk solution (green) or graphene nanochannels (red: mid-position; blue: edge-position) for (a) Li^{+}, (b) Na^{+}, (c) K^{+}, (d) Ca^{2+}, (e) Mg^{2+} or (f) Cl^{-} and corresponding water coordination numbers (N_{W}) versus radial distance (r) from the ion. Simulations were performed with Williams force field. For a given ion, its r_{HS} corresponds to the position of RDF's 1st peak, also shown in Supplementary Table 2; N_{W} for its 1st HS is the N_{W} at the position of RDF's 1st valley. Cl^{-} ion locates at edge-position in graphene 2D nanochannel no matter which FF parameters are used, and thus only RDFs of edge-position are shown; similarly, for Ca^{2+} and Mg^{2+} in nanochannel, only RDFs of mid-position are shown. Source data are provided as a Source Data file.

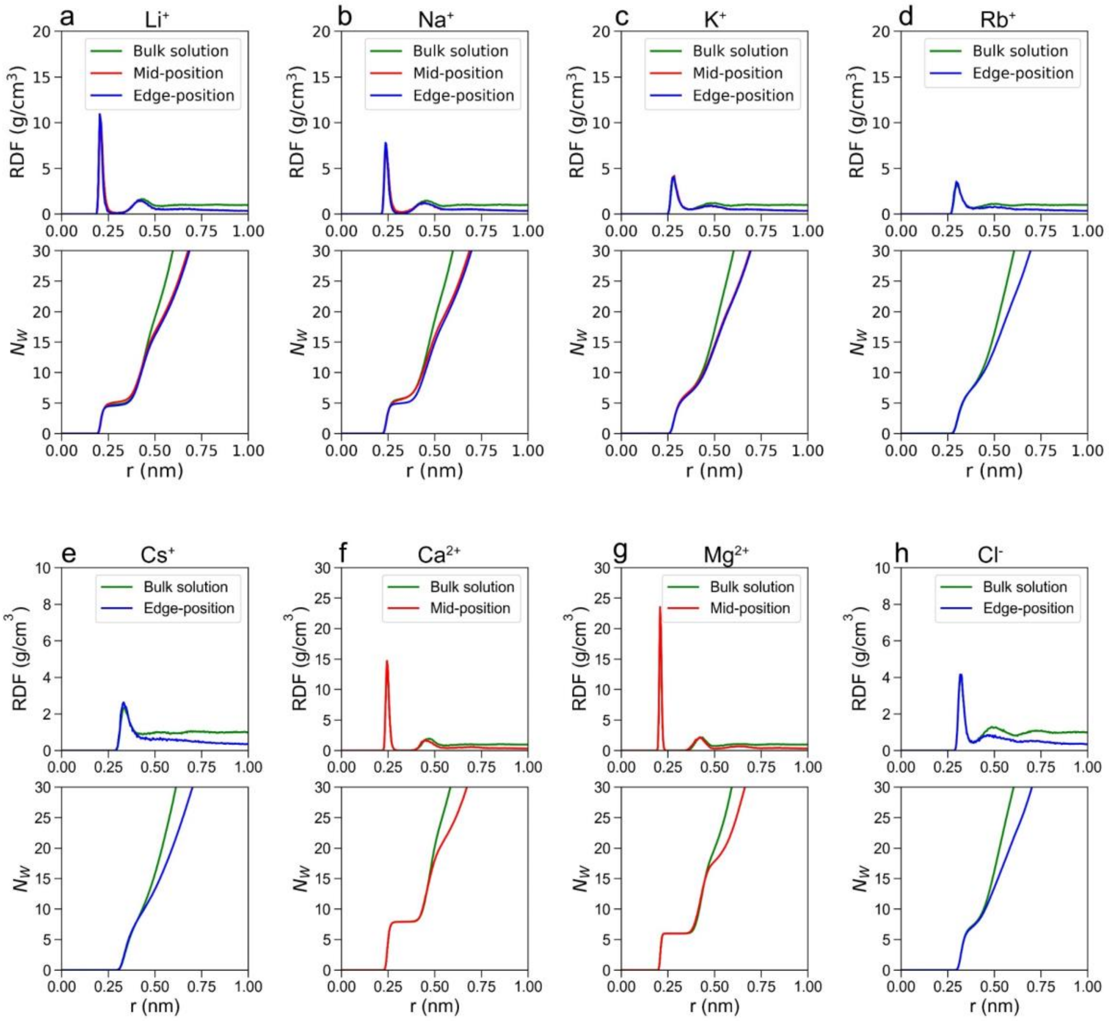

Supplementary Fig. 5 | Radial distribution function (RDF) of ion-(water) oxygen atoms in bulk solution (green) or graphene nanochannels (red: mid-position; blue: edge-position) for (a) Li^+, (b) Na^+, (c) K^+, (d) Rb^+, (e) Cs^+, (f) Ca^{2+}, (g) Mg^{2+} or (h) Cl^- and corresponding water coordination numbers (N_W) versus radial distance (r) from the ion. Simulations were performed with OPLS-AA force field. For a given ion, its r_{HS} corresponds to the position of RDF's 1st peak, also shown in Supplementary Table 2; N_W for its 1st HS is the N_W at the position of RDF's 1st valley. Rb^+, Cs^+ and Cl^- ions locate at edge-position in graphene 2D nanochannel with $\varepsilon_{\mathrm{I-W}}^{\mathrm{LB}}$ parameters used, and thus only RDFs of edge-position are shown; similarly, for Ca^{2+} and Mg^{2+} in nanochannel, only RDFs of mid-position are shown. Source data are provided as a Source Data file.

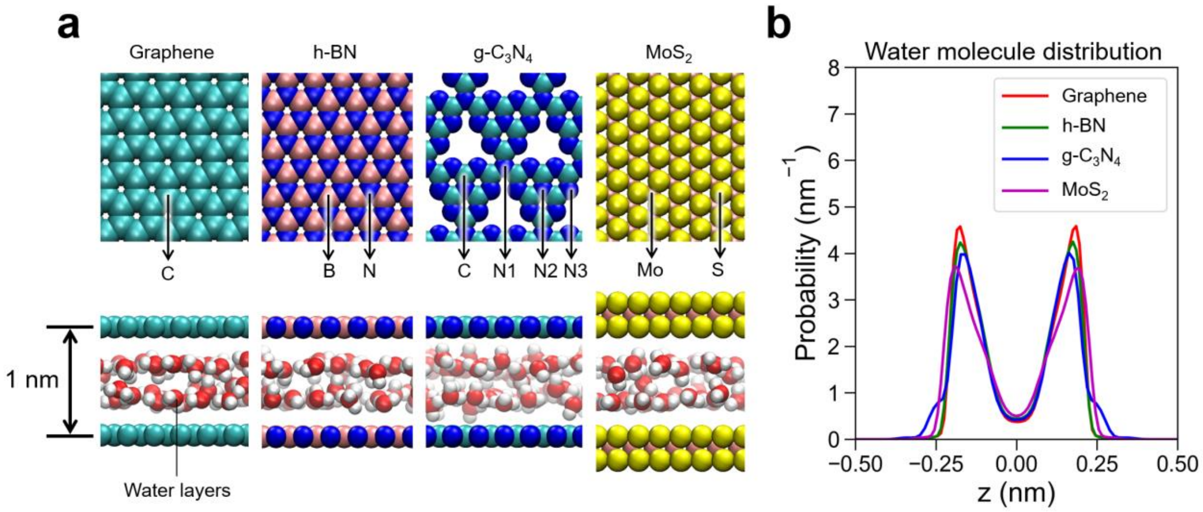

Supplementary Fig. 6 | Distribution profiles of water molecules in 2D nanochannels of graphene, h-BN, g-C_3N_4 or MoS_2. (a) Top views and side views of the four types of 2D nanochannels with the channel height of 1 nm. (b) Water distribution profiles in the four types of 2D nanochannels. Source data are provided as a Source Data file.

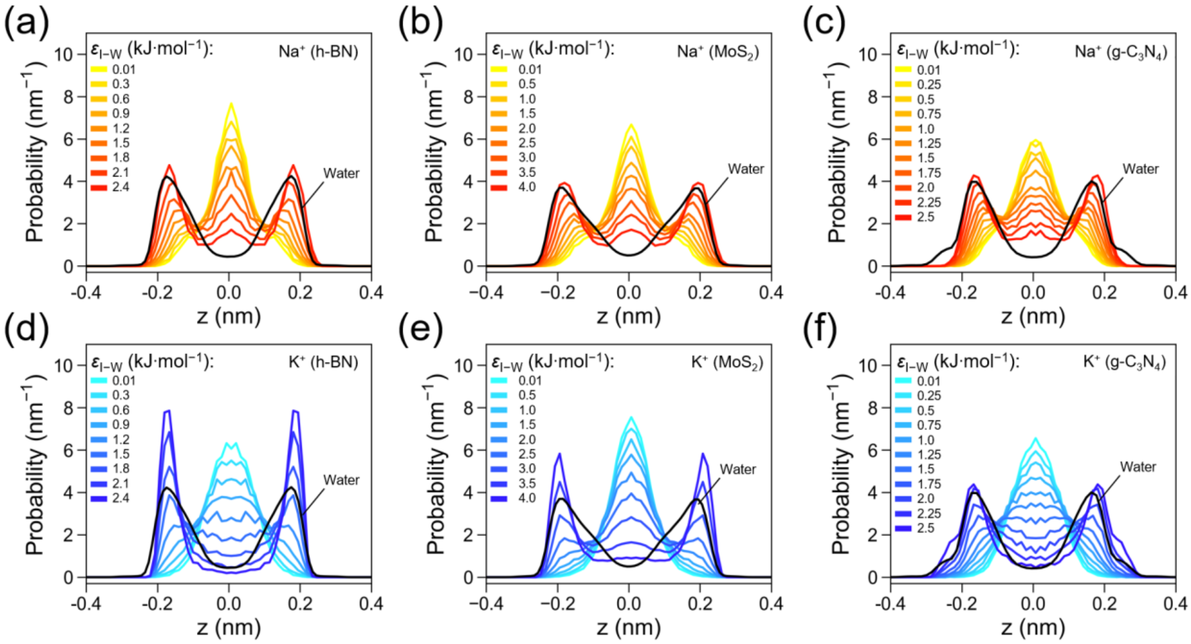

Supplementary Fig. 7 | Distribution profiles of Na^+ (a, b, c) or K^+ (d, e, f) in 2D nanochannel constructed by h-BN (a, d), MoS_2 (b, e) or g-C_3N_4 (c, f), simulated with Williams FF. The LJ parameter between ion and wall atoms ($\varepsilon_{I\text{-}W}$) were adjusted to gradually change $d_{\text{ion-wall}}$, as ions approach water layers with $\varepsilon_{I\text{-}W}$ increasing. Source data are provided as a Source Data file.

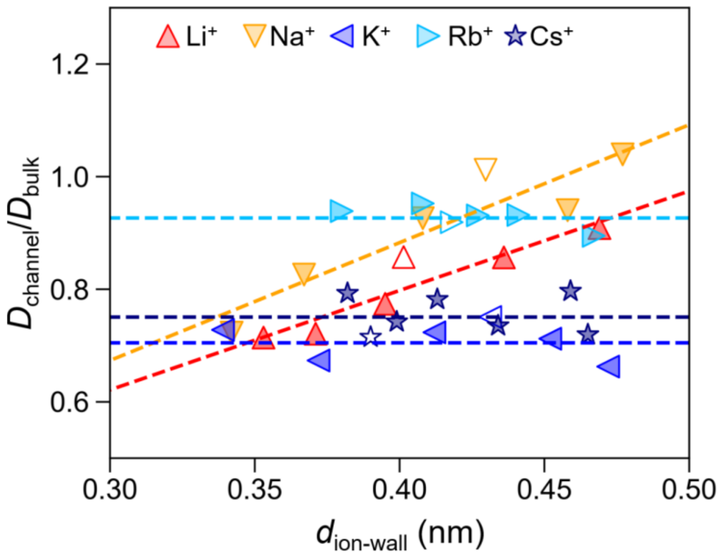

Supplementary Fig. 8 | $D_{channel}/D_{bulk}$ ratio versus $d_{ion\text{-}wall}$ for simulations where ion's z coordinate is restrained by harmonic potential to change $d_{ion\text{-}wall}$ in graphene 2D nanochannel. For ions with small r_{HS} (*e.g.*, Li^+, Na^+), $D_{channel}/D_{bulk}$ ratio linearly corelates with $d_{ion\text{-}wall}$ and the dashed lines are the fitting results for Li^+ or Na^+. For ions with large r_{HS} (*e.g.*, K^+, Rb^+, Cs^+), $D_{channel}/D_{bulk}$ ratio is independent with $d_{ion\text{-}wall}$ and the horizontal lines stand for the mean values of $D_{channel}/D_{bulk}$ results of K^+, Rb^+ or Cs^+. A harmonic potential with a force constant of 2,000 kJ·mol^{-1}·nm^{-2} is applied on the ion in z direction to control $d_{ion\text{-}wall}$. Hollow points are results of simulations without harmonic potential. Simulations were performed with original Merz force field (ε_{I-W}^{LB}). Source data are provided as a Source Data file.

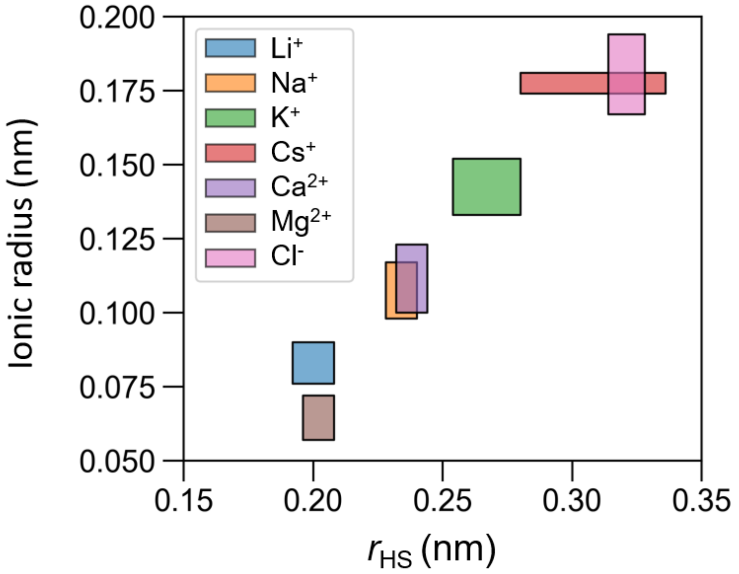

Supplementary Fig. 9 | Ions with larger r_{HS} usually possess larger ionic radius. The length and width of the rectangles stand for the ranges of ionic radius (taken from ref. [13]) and r_{HS} (taken from Supplementary Table 2), respectively. Source data are provided as a Source Data file.

Supplementary Note 1 | How force fields (FFs) and D_{bulk} affect $D_{channel}$.

A close look at $D_{channel}$ (Supplementary Fig. 10) reveals that 1) $D_{channel}$ of Mg^{2+} and Ca^{2+} are usually larger than corresponding D_{bulk}, while $D_{channel}$ of other ions are usually smaller; 2) When comparing the original version of FF (ε_{I-W}^{LB}) with the optimized one ($\varepsilon_{I-W}^{ion-\pi}$), $D_{channel}$ of Na^{+} show the most significant change with corresponding distribution profiles also changing greatly (Supplementary Fig. 1); 3) D_{bulk} affects $D_{channel}$ to some extent, *i.e.* larger D_{bulk} usually leads to larger $D_{channel}$. Although $D_{channel}$ themselves reveal less information than $D_{channel}/D_{bulk}$, they yield some useful clues as follows. The above point 2 inspires us consider $d_{ion\text{-}wall}$, which describes the change of ion distribution profiles. Point 3 inspires us study the ratio $D_{channel}/D_{bulk}$, so that we could focus on the difference between nanochannel and bulk solution and how such difference affect the motion mechanism for various ions (the main point of our work), instead of discuss specific details of individual FFs or ions. In this way, all the studied ions and FFs in our work could complete the puzzle, '$D_{channel}/D_{bulk}$ of small ions (r_{HS} < K^{+}'s r_{HS}) increases with $d_{ion\text{-}wall}$, while that of bulky ions ($r_{HS} \geq K^{+}$'s r_{HS}) is independent on $d_{ion\text{-}wall}$' (Fig.1b).

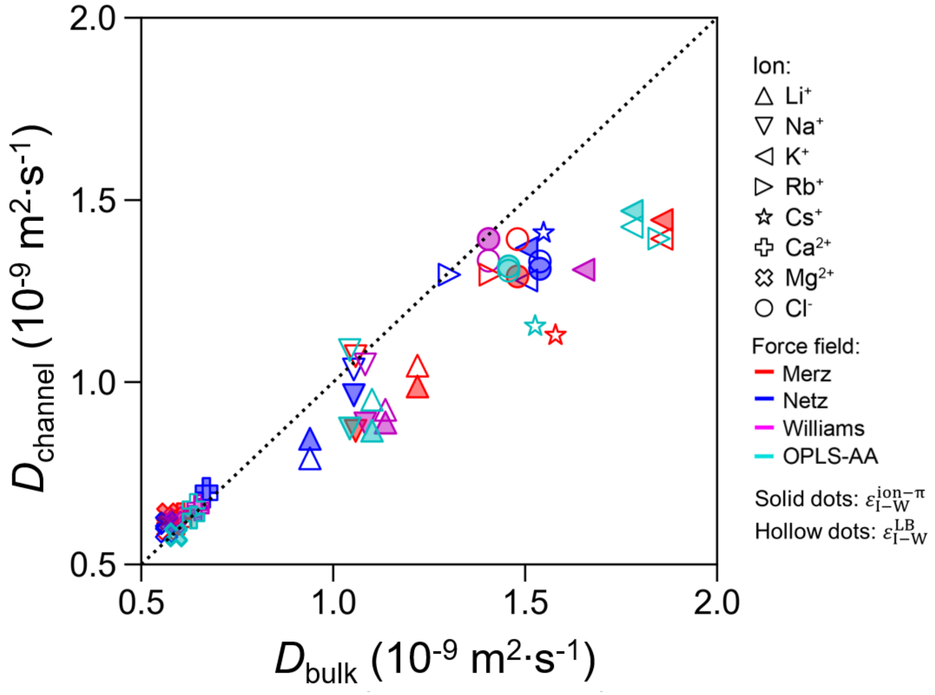

Supplementary Fig. 10 | $D_{channel}$ in graphene 2D nanochannel versus D_{bulk} for the studied ions simulated with different force fields. For each force field, two different sets of LJ parameters between ion and graphene, $\varepsilon_{I-W}^{ion-\pi}$ and ε_{I-W}^{LB} (see Supplementary Table 2), were employed. The dot line is the guide line of y = x. Source data are provided as a Source Data file.

Supplementary Note 2 | Ion-graphene interaction hardly affects ion's diffusivity.

Na^+ and K^+, z coordinates fixed either at mid-position ($d_{ion\text{-}wall}$ = 0.5 nm) or edge-position (peak position of the distribution profile with $d_{ion\text{-}wall}$ < 0.5 nm, see Fig. 1a), were simulated with $\varepsilon_{I\text{-}W}$ varying, and the resulting $D_{channel}/D_{bulk}$ is constant (Supplementary Fig. 11) even when $\varepsilon_{I\text{-}W}$ = 8 kJ/mol (~4 times of $\varepsilon_{I-W}^{ion-\pi}$). This indicates ion-graphene interaction hardly affects $D_{channel}$ in the studied $\varepsilon_{I\text{-}W}$ range, in other words, the $D_{channel}/D_{bulk} \sim d_{ion\text{-}wall}$ correlation could be attributed to the interaction between ion and water layers, as the system only consists of ion, graphene and water, which forms 2 layers in the studied nanochannel.

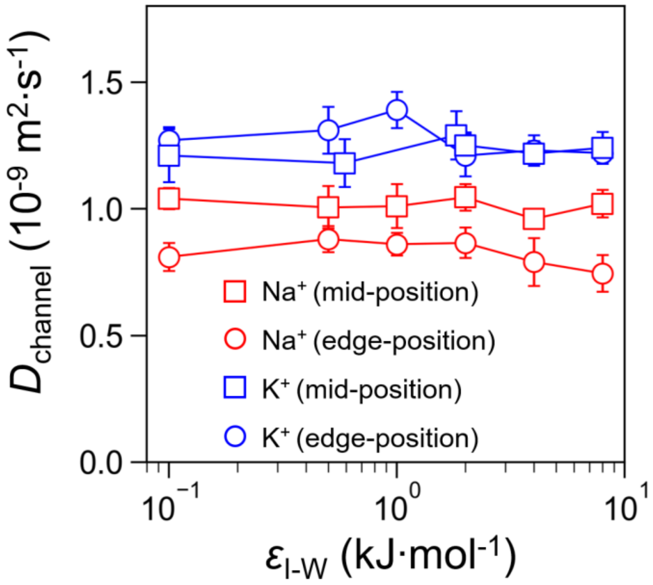

Supplementary Fig. 11 | The simulated *D* of Na^+ and K^+ ions with z coordinates fixed either at mid-position or edge-position, and $\varepsilon_{I\text{-}W}$ varying. K^+ (blue) and Na^+ (red) at mid-position (square) or edge-position (circle) were simulated with the Merz force field. Note $D_{channel}$ in this figure might be somewhat different from those in Supplementary Table 3, as fixing the z coordinate of ions may affect their diffusivity[14]. Error bars represent the standard error (n = 3 independent MD simulations), and the centers of error bars indicate the means. Source data are provided as a Source Data file.

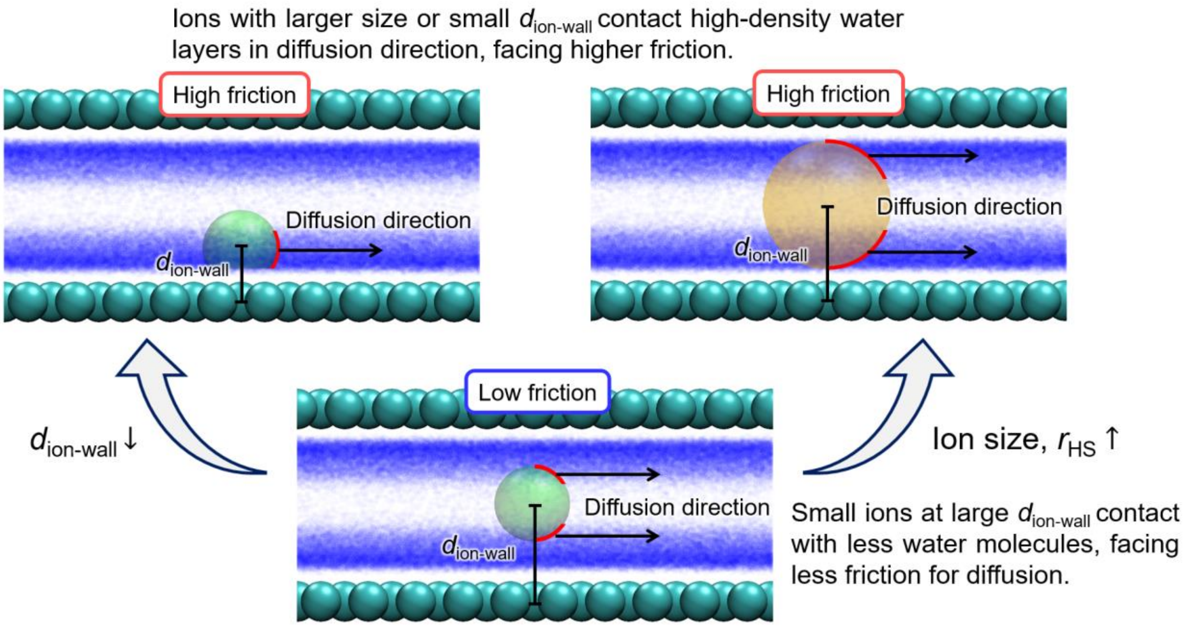

Supplementary Fig. 12 | Ions with small r_{HS} and large $d_{ion\text{-}wall}$ face less friction for diffusion (bottom), while ions with larger r_{HS} or small $d_{ion\text{-}wall}$ suffer from larger friction from water layers for diffusion (upper). The green and yellow balls stand for the 1st hydration shell for small ions and large ions, respectively. The red curves stand for the contact area (towards diffusion direction) between ion's 1st hydration and water layers. The blue area stands for water layers, with darker colors indicating higher water density.

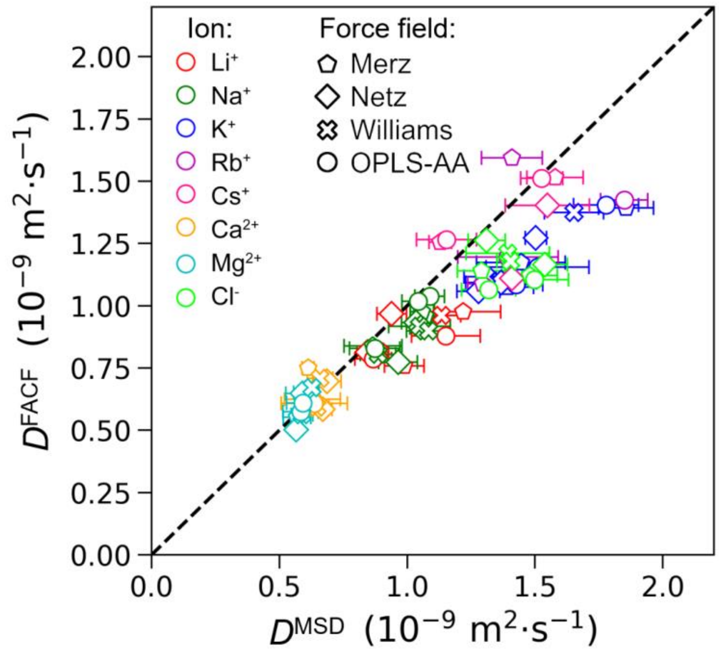

Supplementary Fig. 13 | Ions' diffusivities obtained by FACF method (D^{FACF}) are quite consistent with those obtained by MSD method (D^{MSD}). See 'Method' section for calculation details of these two methods. The dashed line is the guide line of y = x. It is worthy pointing out that, D^{FACF} sometimes may deviate from D^{MSD} due to the effect of fixing the ion's position when we calculate FACF[15,16]. That is, fixing the ion's position changes the water exchange dynamics around as the ion could not move in response to the hydration water dynamics[15]. Nevertheless, such deviation may not affect the following discussions on friction change, as we discuss $D_{\text{channel}}/D_{\text{bulk}}$ instead of D_{channel}, and $D^{\text{FACF}}_{\text{channel}}/D^{\text{FACF}}_{\text{bulk}} \sim d_{\text{ion-wall}}$ and $D^{\text{MSD}}_{\text{channel}}/D^{\text{MSD}}_{\text{bulk}} \sim d_{\text{ion-wall}}$ follow similar rule (see below Supplementary Fig. 14). Error bars represent the standard error (n = 3 independent MD simulations), and the centers of error bars indicate the means. Source data are provided as a Source Data file.

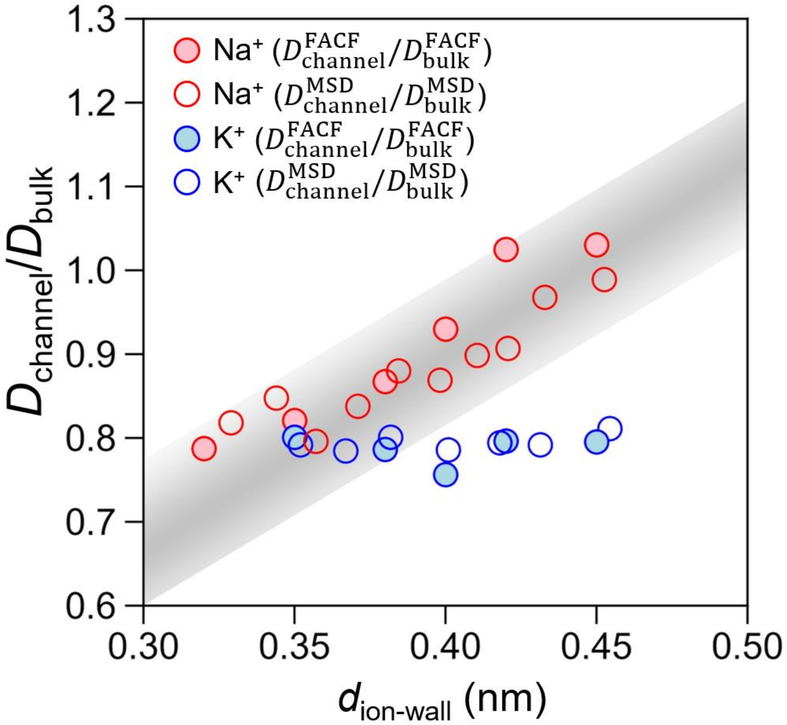

Supplementary Fig. 14 | $D^{FACF}_{channel}/D^{FACF}_{bulk}$ ~ $d_{ion\text{-}wall}$ and $D^{MSD}_{channel}/D^{MSD}_{bulk}$ ~ $d_{ion\text{-}wall}$ follow similar rule. The shadowed area is the prediction error of $D_{channel}/D_{bulk}$ ~ $d_{ion\text{-}wall}$ linear correlation, identical with that in Fig. 1b. $D^{FACF}_{channel}$ and D^{FACF}_{bulk} are diffusivities calculated from force-autocorrelation function simulations which fixed the ion at different positions in the nanochannel or in bulk solution respectively. The data of $D^{MSD}_{channel}/D^{MSD}_{bulk}$ (the superscript MSD indicates the diffusivity data are calculated from the MSD) are taken from Fig. 1d. Although D^{FACF} sometimes deviates from D^{MSD} (Supplementary Fig. 13), $D^{FACF}_{channel}/D^{FACF}_{bulk}$ ~ $d_{ion\text{-}wall}$ and $D^{MSD}_{channel}/D^{MSD}_{bulk}$ ~ $d_{ion\text{-}wall}$ follow similar rule, as the fixing position (confinement) effect[15,16] in nanochannel cancels with that in bulk solution to a large extent. Source data are provided as a Source Data file.

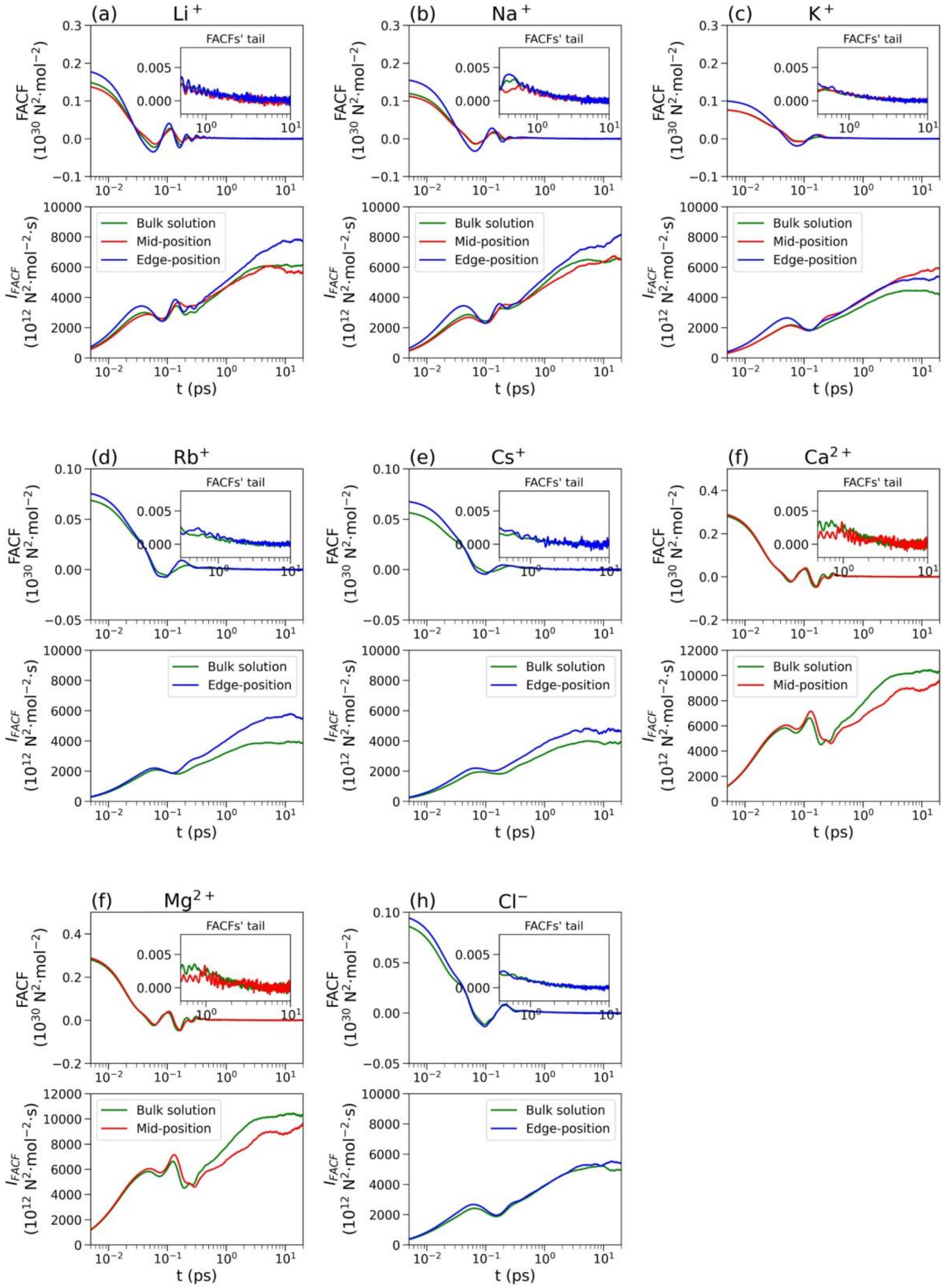

Supplementary Fig. 15 | FACF (top) and I_{FACF} (bottom) of (a) Li$^+$, (b) Na$^+$, (c) K$^+$, (d) Rb$^+$, (e) Cs$^+$, (f) Ca^{2+}, (g) Mg^{2+} or (h) Cl$^-$ in bulk solution (green) or 2D nanochannels (red: mid-position; blue: edge-position). The insets are the FACFs' tail parts where FACFs slowly decay to 0. Simulations were performed with Merz force field. Source data are provided as a Source Data file.

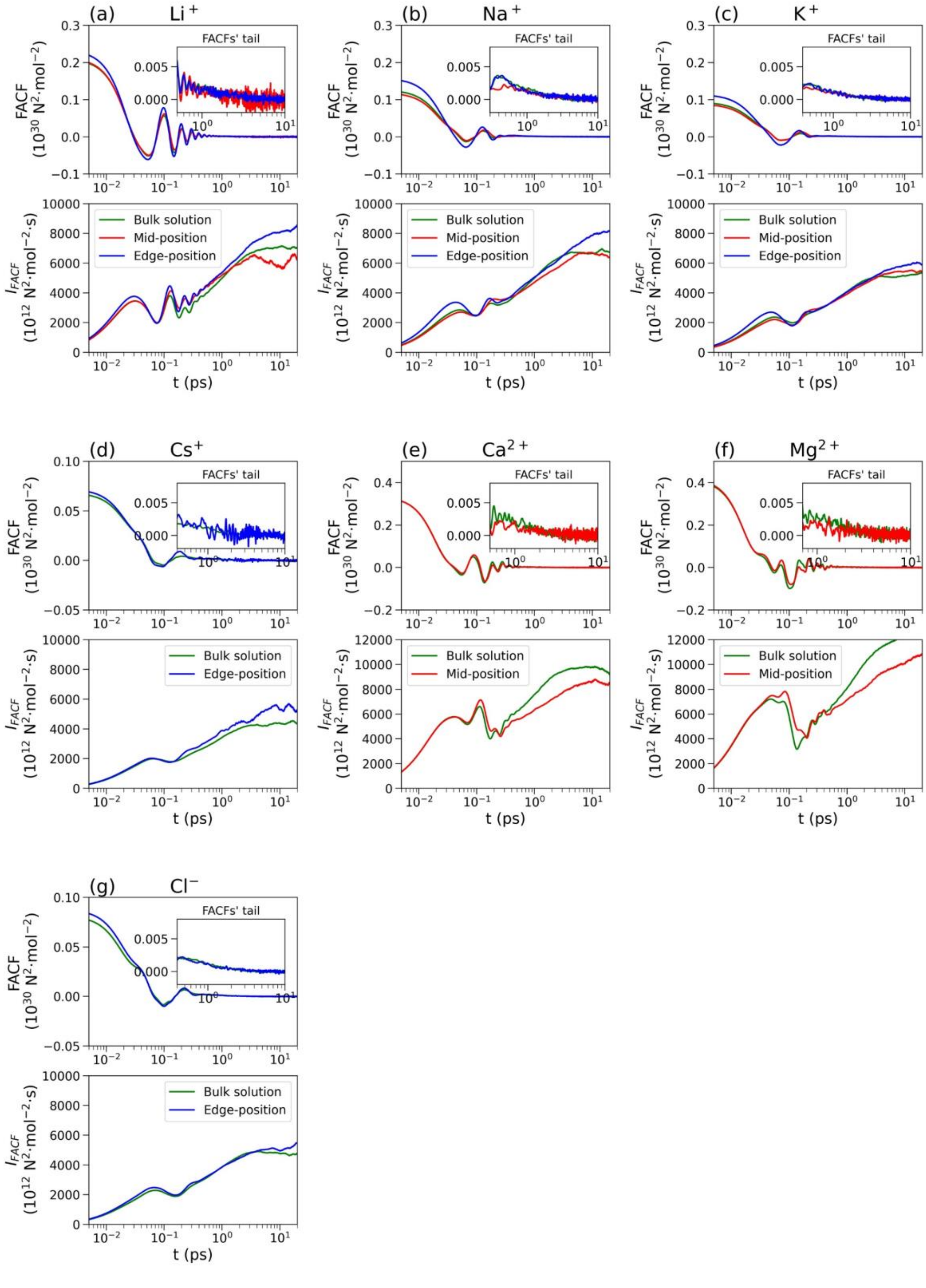

Supplementary Fig. 16 | FACF (top) and I_{FACF} (bottom) of (a) Li^{+}, (b) Na^{+}, (c) K^{+}, (d) Cs^{+}, (e) Ca^{2+}, (f) Mg^{2+} or (g) Cl^{-} in bulk solution (green) or 2D nanochannels (red: mid-position; blue: edge-position). The insets are the FACFs' tail parts where FACFs slowly decay to 0. Simulations were performed with Netz force field. Source data are provided as a Source Data file.

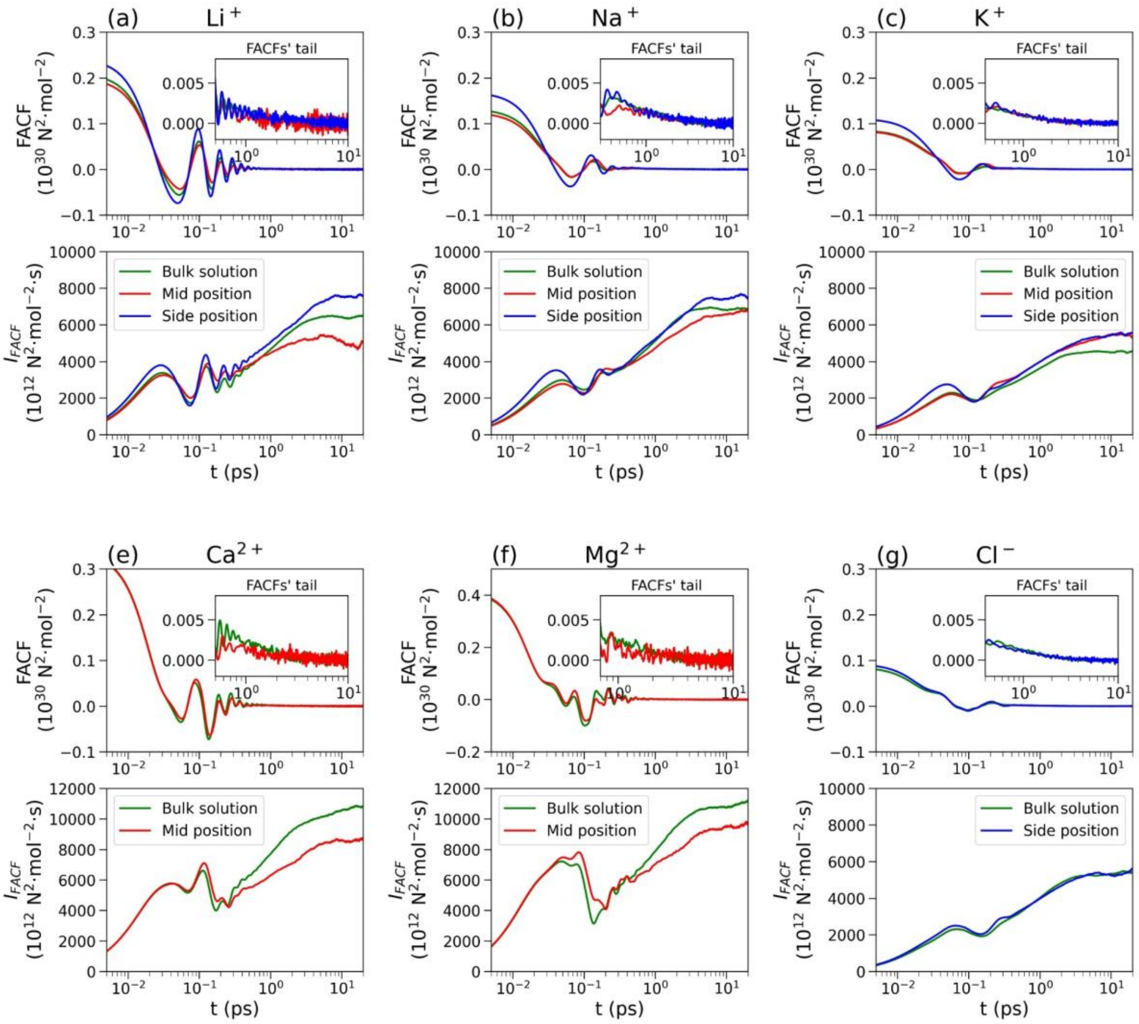

Supplementary Fig. 17 | FACF (top) and I_{FACF} (bottom) of (a) Li^+, (b) Na^+, (c) K^+, (d) Ca^{2+}, (e) Mg^{2+} or (f) Cl^- in bulk solution (green) or 2D nanochannels (red: mid-position; blue: edge-position). The insets are the FACFs' tail parts where FACFs slowly decay to 0. Simulations were performed with Williams force field. Source data are provided as a Source Data file.

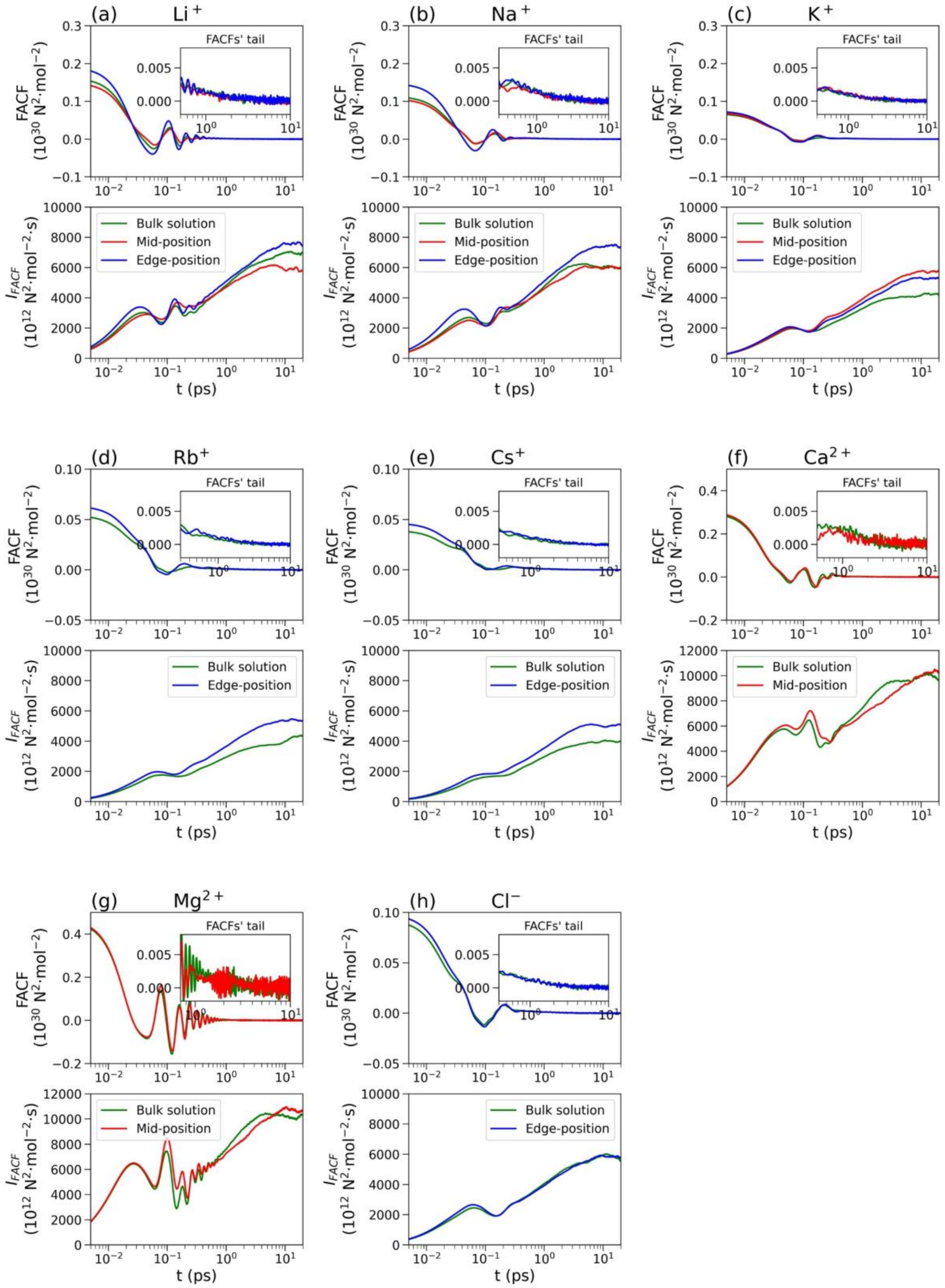

Supplementary Fig. 18 | FACF (top) and *I*FACF (bottom) of (a) Li^+, (b) Na^+, (c) K^+, (d) Rb^+, (e) Cs^+, (f) Ca^{2+}, (g) Mg^{2+} or (h) Cl^- in bulk solution (green) or 2D nanochannels (red: mid-position; blue: edge-position). The insets are the FACFs' tail parts where FACFs slowly decay to 0. Simulations were performed with OPLS-AA force field. Source data are provided as a Source Data file.

Supplementary Table 4 | I_{FACF}, I_{head}, I_{tail} and τ of ions in different solvation environments (bulk, nanochannels' mid and edge positions) when simulated with Merz FF.

Ions	Position	I_{FACF} (10^{12} $N^2 \cdot mol^{-2} \cdot s$)	I_{head} (10^{12} $N^2 \cdot mol^{-2} \cdot s$)	I_{tail} (10^{12} $N^2 \cdot mol^{-2} \cdot s$)	τ (ps)
Li^+	Bulk	6292.1	3914.6	2377.5	66.4
	Mid	5781.3	4057.1	1724.2	96.5
	Edge	8087.8	4222.4	3865.4	294.3
Na^+	Bulk	6285.6	3356.6	2929.0	57.8
	Mid	6399.8	3648.8	2751.0	106.3
	Edge	7321.9	3500.3	3821.6	602.2
K^+	Bulk	4409.3	2313.7	2095.6	20.7
	Mid	5706.7	2712.8	2993.9	82.7
	Edge	5231.8	2551.9	2679.9	74.3
Rb^+	Bulk	3853.3	2342.5	1510.8	12.24
	Mid	-	-	-	-
	Edge	5637.8	2849.1	2788.7	28.72
		-	-	-	-
Cs^+	Bulk	4051.6	2446.0	1605.6	10.89
	Mid	-	-	-	-
	Edge	4890.2	2905.7	1984.4	21.49
Ca^{2+}	Bulk	10446.5	6502.4	3944.1	799.2
	Mid	8191.6	5857.5	2334.1	2182.5
	Edge	-	-	-	-
Mg^{2+}	Bulk	11095.6	7641.4	3454.2	∞
	Mid	9422.9	6764.6	2658.3	∞
	Edge	-	-	-	-
Cl^-	Bulk	5471.3	2984.6	2486.7	62.9
	Mid	-	-	-	-
	Edge	5381.6	3070.2	2311.4	109.8

Supplementary Table 5 | I_{FACF}, I_{head}, I_{tail} and τ of ions in different solvation environments (bulk, nanochannels' mid and edge positions) when simulated with Netz FF.

Ion	Position	I_{FACF} (10^{12} $N^2 \cdot mol^{-2} \cdot s$)	I_{head} (10^{12} $N^2 \cdot mol^{-2} \cdot s$)	I_{tail} (10^{12} $N^2 \cdot mol^{-2} \cdot s$)	τ (ps)
Li^+	Bulk	6342.4	3718.5	2623.9	611.2
	Mid	5468.8	4427.3	1041.5	537.9
	Edge	7538.8	4259.0	3279.8	3487.8
Na^+	Bulk	6731.9	3315.2	3416.7	56.3
	Mid	6581.3	3489.6	3091.7	105.9
	Edge	7938.4	3510.4	4428.0	457.8
K^+	Bulk	4822.3	2636.5	2185.8	29.1
	Mid	5804.5	2892.1	2912.4	75.4
	Edge	5612.1	2708.3	2903.8	92.0
Cs^+	Bulk	4375.7	2350.4	2025.3	12.5
	Mid	-	-	-	-
	Edge	5526.7	2650.3	2876.4	28.3
Ca^{2+}	Bulk	10500.0	6331.7	4168.3	415.2
	Mid	8814.9	5587.8	3227.1	431.7
	Edge	-	-	-	-
Mg^{2+}	Bulk	12216.5	7876.5	4340.0	∞
	Mid	9481.8	6955.9	2525.9	∞
	Edge	-	-	-	-
Cl^-	Bulk	5269.1	2982.9	2286.2	27.8
	Mid	-	-	-	-
	Edge	4862.4	3028.7	1833.7	62.6

Supplementary Table 6 | I_{FACF}, I_{head}, I_{tail} and τ of ions in different solvation environments (bulk, nanochannels' mid and edge positions) when simulated with Williams FF.

Ion	Position	I_{FACF} (10^{12} $N^2 \cdot mol^{-2} \cdot s$)	I_{head} (10^{12} $N^2 \cdot mol^{-2} \cdot s$)	I_{tail} (10^{12} $N^2 \cdot mol^{-2} \cdot s$)	τ (ps)
Li^+	Bulk	6391.9	3688.3	2703.6	106.4
	Mid	5212.6	3939.5	1273.1	109.3
	Edge	7548.2	4154.1	3394.1	514.7
Na^+	Bulk	6824.8	3499.7	3325.1	61.2
	Mid	6693.3	3700.9	2992.4	116.1
	Edge	7615.2	3482.8	4132.4	913.6
K^+	Bulk	4467.6	2418.3	2049.3	22.3
	Mid	5513.6	2876.4	2637.2	80.9
	Edge	5496.7	2573.8	2922.9	82.8
Ca^{2+}	Bulk	10364.3	6285.9	4078.4	405.7
	Mid	8662.6	5791.2	2871.4	442.6
	Edge	-	-	-	-
Mg^{2+}	Bulk	10905.7	7678.2	3227.5	∞
	Mid	9136.3	6640.2	2496.1	∞
	Edge	-	-	-	-
Cl^-	Bulk	5210.4	2921.0	2289.4	33.4
	Mid	-	-	-	-
	Edge	5070.4	3034.2	2036.2	53.2

Supplementary Table 7 | I_{FACF}, I_{head}, I_{tail} and τ of ions in different solvation environments (bulk, nanochannels' mid and edge positions) when simulated with OPLS-AA FF.

Ions	Position	I_{FACF} (10^{12} $N^2 \cdot mol^{-2} \cdot s$)	I_{head} (10^{12} $N^2 \cdot mol^{-2} \cdot s$)	I_{tail} (10^{12} $N^2 \cdot mol^{-2} \cdot s$)	τ (ps)
Li^+	Bulk	6983.0	3513.1	3469.9	90.8
	Mid	5829.9	3687.1	2142.8	133.8
	Edge	7803.4	3806.5	3996.9	227.0
Na^+	Bulk	6031.2	3478.4	2552.8	55.1
	Mid	5910.6	3599.3	2311.3	128.8
	Edge	7406.2	3769.1	3637.1	429.5
K^+	Bulk	4370.8	2719.6	1650.2	29.1
	Mid	5662.2	3094.4	2567.8	108.8
	Edge	5316.7	2988.2	2328.5	92.4
Rb^+	Bulk	4306.6	2009.3	2297.3	9.71
	Mid	-	-	-	-
	Edge	5134.9	2509.3	2625.6	21.58
Cs^+	Bulk	4059.4	2182.2	1877.2	6.24
	Mid	-	-	-	-
	Edge	4850.2	2590.6	2259.6	18.85
Ca^{2+}	Bulk	10095.4	6767.2	3328.2	761.2
	Mid	9832.9	5989.1	3843.8	1742.9
	Edge	-	-	-	-
Mg^{2+}	Bulk	10071.0	6766.3	3304.7	∞
	Mid	10680.1	6572.2	4107.9	∞
	Edge	-	-	-	-
Cl^-	Bulk	5551.7	3013.9	2537.8	56.7
	Mid	-	-	-	-
	Edge	5763.7	2939.2	2824.5	102.6

Supplementary Note 3 | The change of I_{FACF} mainly comes from I_{tail}, whereas I_{head} hardly changes for a given ion in different solvation environments.

The FACF curves (Supplementary Figs. 15-18) usually violently oscillate when $t < \sim 0.3$ ps (the head part), possessing a series of positive or negative parts. When comparing an ion locating in different solvation environment, a more positive part in FACF's head is usually followed by a more negative part (Supplementary Figs. 15-18), thus their contributions to I_{head} cancel with each other (a simple explanation is that, more positive FACF = <*F*(*t*)·*F*(0)> indicates large force between ion and water, which may also yield more negative <*F*(*t*)·*F*(0)> for other t). Consequently, I_{head} of an ion locating in different solvation environment are usually quite similar (Supplementary Figs. 15-18). To the contrary, though FACFs' tail parts are close to 0 (Supplementary Figs. 15-18), they last more than 9 ps (dozens of times longer than FACF's head parts) and thus, their change dominates the change of ΔI_{FACF} (comparing the same ion in different solvation environments), in other words, $\Delta I_{\mathrm{tail}} \approx \Delta I_{\mathrm{FACF}}$ (Fig. 2c).

Supplementary Note 4 | When an ion approaches water layers or possesses large r_{HS}, the nanoconfinement effect would overwhelm the z (channel-height or d-spacing direction) component water-ion interactions to compress the ion's HS and thus increase HS density (ρ_{HS}).

When an ion locates in bulk solution, the water-ion interactions (WIs) of spherical symmetry yield a spherical HS for the ion (left column of Fig. 3b). However, when it locates in the graphene nanochannel, the nanoconfinement effect also shapes its HS. The nanoconfinement effect is the driving force for water layers forming in the nanochannel, in other words, it drives water molecules in the middle of nanochannel (mid-position) to graphene surfaces (edge-position). Thus, it could be taken as some kind of 'force', named as 'nanoconfinement force' (NCF), which includes the graphene-water interactions and water-water interactions. For different ions confined in a nanochannel, the NCF is determined by the nanochannel, thus its effect on the HSs is similar. However, WIs for different ions are different. The HS of ion in graphene nanochannel is shaped by both WI and NCF (Figs. 3a&b, Supplementary Fig. 19). As for ions with small r_{HS} (*e.g.* Li^+, Na^+), their WIs are quite strong and have predominant effect on their HSs. Yet, for ions with large r_{HS} (*e.g.* K^+), their WIs are weaker and thus the NCF dominates their HSs. The detailed explanations are as follows:

1. When ions locate at mid-position

For an ion with small r_{HS}, *e.g.* Li^+ or Na^+, its WI is quite large due to the small ion-water distance (Supplementary Table. 2, Supplementary Fig. 20) and overwhelm the NCF. Thus, its HS at mid-position (HS_{mid}) is mainly shaped by the WI. Consequently, its HS_{mid} is spherical (Supplementary Fig. 19), similar to that in bulk solution (HS_{bulk}), as WI shows spherical symmetry. Thus, for ions with small r_{HS} (*e.g.* Li^+, Na^+), the densities of corresponding HSs are also similar:

$$\rho_{\mathrm{HS}}^{\mathrm{mid}} \approx \rho_{\mathrm{HS}}^{\mathrm{bulk}} \tag{S1}$$

where $\rho_{\mathrm{HS}}^{\mathrm{mid}}$ is HS_{mid}'s density and $\rho_{\mathrm{HS}}^{\mathrm{bulk}}$ is HS_{bulk}'s density.

For an ion with large r_{HS}, *e.g.* K^+, its WI becomes weaker (Supplementary Fig. 20). Thus, NCF (yielding two water layers) overwhelms the z component of WI (yielding a spherical HS). In other words, NCF plays a predominant role on shaping its HS. Consequently, the HS is distorted: water molecules in the HS are driven to the edge position, with the HS compressed to two rings (Supplementary Fig. 19). As the volume of rings is smaller than that of spherical HS (note the rings are just part of the HS sphere), and the water coordination number (N_W) of K^+ in mid position is close to that in bulk solution (Supplementary Figs. 2-5). Thus, for ions with large r_{HS} (*e.g.* K^+), we obtain:

$$\rho_{\mathrm{HS}}^{\mathrm{mid}} > \rho_{\mathrm{HS}}^{\mathrm{bulk}} \tag{S2}$$

Note ρ_{HS} is the localized nuclear density of water molecules (there is void surrounding the HS, as indicated by the high free energy region in Fig. 3b), thus could be quite large, *e.g.* the max ρ_{HS} of K^+'s HS (simulated with Merz FF) in bulk water is 18.2 g/cm^3, while that of Na^+'s HS is 27.2 g/cm^3 (see Fig. 2b and equation 8 in Methods section). It is to the contrary of density of bulk water (ρ_{bulk}), which is a uniform continuum, and a large ρ_{bulk} usually indicates huge pressure.

2. When ions locate at edge-position

For an ion with small r_{HS}, *e.g.* Li^+ or Na^+, ~ ½ of its hydration sphere is no longer available for water molecules due to the steric hindrance of the channel wall[12], yet its N_W is very similar to N_W in bulk solution (Supplementary Figs. 2-5). This indicates its ρ_{HS} would increase considerably than ρ_{HS} in bulk solution, in other words, the HS of Li^+ or Na^+ at edge-position (HS_{edge}) would be quite crowded. Consequently, the pole and ring parts of HS_{edge} (Figs. 3a&3b), corresponding to the two water layers, could accommodate water molecules, since these two positions are favored by both NCF (yielding two water layers) and WIs (yielding a spherical HS). The space between the pole and the ring (Fig. 3a), corresponding to the middle of nanochannel, however, could no longer accommodate water, because water molecules in the pole and ring parts would repel any other water molecules between these two regions. Such case is to the sharp contrary of the ion locating at mid-position, where the HS is quite spherical as there is no steric hindrance of the channel wall to distort the ion's HS. Consequently, the shape of HS_{mid} is close to that of HS_{bulk}, yielding $\rho_{HS}^{mid} \approx \rho_{HS}^{bulk}$ (note N_w for ion in 2D nanochannel is similar to that in bulk solution, as Supplementary Figs. 2-5 indicate). Overall, for ions with small r_{HS} (*e.g.* Li^+, Na^+), we obtain:

$$\rho_{HS}^{edge} > \rho_{HS}^{mid} \approx \rho_{HS}^{bulk} \tag{S3}$$

where ρ_{HS}^{edge} is HS_{edge}'s density. Notably, water density slightly fluctuates in different parts of HS in nanochannels. The water density in the ring parts of the HS (the space in directions parallel to the surfaces, see Fig. 3a) is a bit larger than ρ_{HS}^{bulk} (accommodate more water molecules), while in other parts is slightly smaller. We did not discuss such fluctuation further, as it does not directly affect ions' diffusivity.

For an ion with large r_{HS}, *e.g.* K^+, its HS_{edge} was also compressed to a pole and a ring (see above discussions). Nevertheless, on one hand, its HS_{mid} is significantly compressed to two rings with the volume not very higher than that of HS_{edge}. On the other hand, the N_W of K^+ at edge-position drops by ~ 1 than that at edge position (Supplementary Figs. 2-5), which decreases ρ_{HS}^{edge}. Consequently, for K^+, the density of HS_{edge} is similar to that of HS_{mid}, $\rho_{HS}^{edge} \approx \rho_{HS}^{mid}$. For ions with large r_{HS} (*e.g.* K^+), we obtain:

$$\rho_{HS}^{edge} \approx \rho_{HS}^{mid} > \rho_{HS}^{bulk} \tag{S4}$$

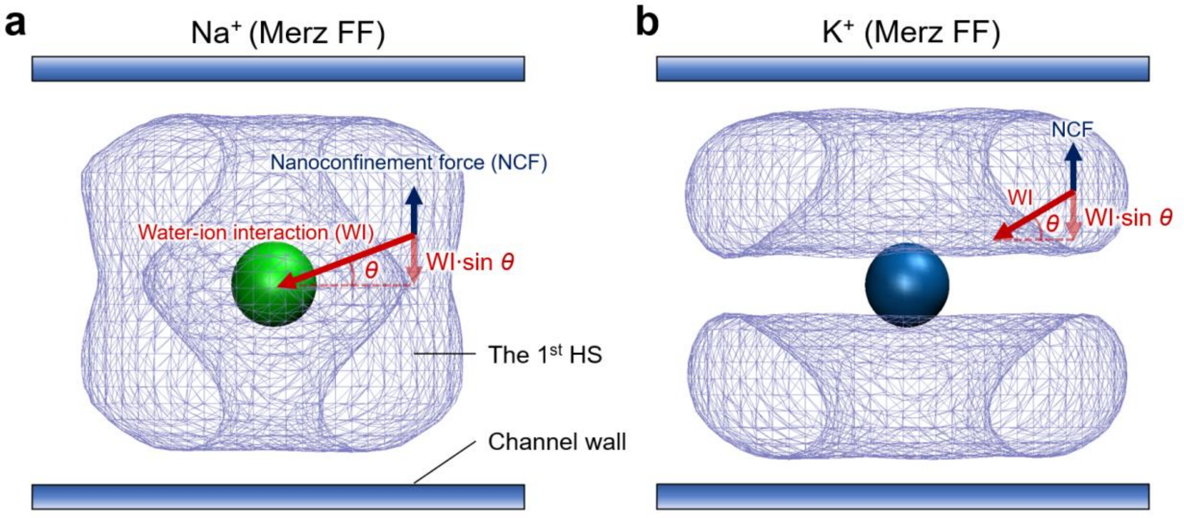

Supplementary Fig. 19 | HS_{mid} of (a) Na^+ or (b) K^+ shaped by both WI and NCF. The purple meshes are the spatial distribution functions (SDFs) for water molecules in the 1st HS (isosurface: 1 g/cm^3). The direction of NCF is along the channel height, while that of WI is from water molecule pointing to the ion (spherical symmetry), as explained in the Supplementary Note 4. The effect of NCF on HS could be significant, and the reason could be explained as follows. As for water molecules located near the middle of nanochannel, the angle (θ) between their WI (with the ion) and the channel wall is quite small. In addition, WI of K^+ with water in HS is not strong (Supplementary Fig. 20). Thus the z component of K^+'s WI, WI·sin θ, is even small. Consequently, NCF would exceed K^+'s WI·sin θ for water molecules at mid position, and drives them to approach the channel wall, until the angle (θ) increases and WI·sin θ could resist NCF. Consequently, water molecules in K^+'s HS would not locate at mid position (small θ), and the HS splits into 2 rings (a). To the contrary, Na^+'s WI with water molecules in HS is quite strong (Supplementary Fig. 20). Thus the z component, WI·sin θ could resist NCF, even for small θ. That is, water molecules in Na^+'s HS could locate near the middle of nanochannel (small θ), thus the HS is close to a sphere, similar like HS in bulk water (a).

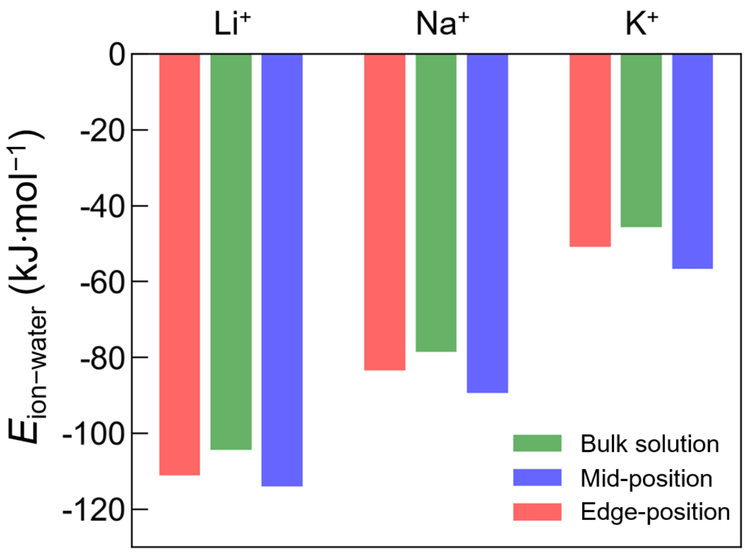

Supplementary Fig. 20 | The interaction energy between ion and water molecules in the 1st HS ($E_{ion\text{-}water}$), simulated with Merz FF. Source data are provided as a Source Data file.

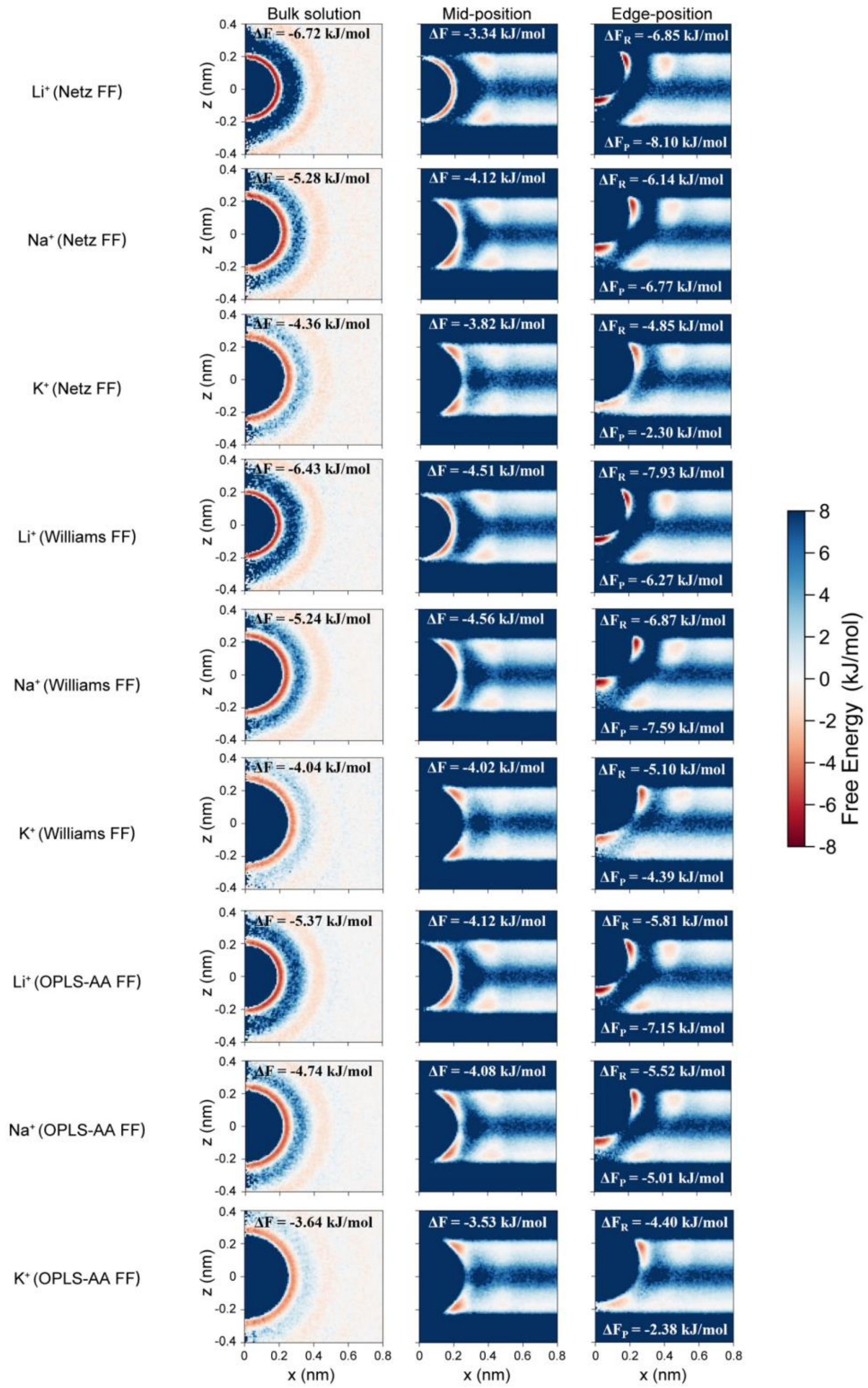

Supplementary Fig. 21 | PMF profiles of Li^+, Na^+ and K^+ ions in bulk solution (left column), channels' mid position (middle column) and edge position (right column) when simulated with Netz FF (1st ~ 3rd rows), Williams FF (4th ~ 6th rows) or OPLS-AA FF (7th ~ 9th rows). The results of Merz FF are shown in Fig. 3b of the main text. Source data are provided as a Source Data file.

Supplementary Note 5 | Validation of the Nernst–Einstein relation for ions in nanochannels through both theoretical derivation and MD simulation data.

Under a uniform electric field, an ion experiences an electric field force:

$$F_e = E\cdot q \tag{S5}$$

Where E is the strength of electric field and q is ion's charge. When the ion moves (driven by F_e) through the solvent at a constant speed, F_e could be balanced by another frictional force (F_{fric}) in opposite direction:

$$F_e = F_{fric} \tag{S6}$$

$$F_{fric} = \lambda_{i\text{-}w}\cdot v \tag{S7}$$

where $\lambda_{i\text{-}w}$ is ion-water friction coefficient and v is ion's speed. Equation. S7, *i.e.*, the Stokes equation, is exact when v is not very high (in the linear regime). We then recall the Einstein equation, which is exact no matter in bulk solution or in nanochannel:

$$D = \frac{k_B T}{\lambda_{i-w}} \tag{S8}$$

where D is ion's diffusivity, k_B is Boltzmann constant and T is temperature. Combining equations S5-S8, we obtain:

$$D = \frac{k_B T}{\lambda_{i-w}} = \frac{k_B T v}{F_{fric}} = \frac{k_B T v}{q\cdot E} = \frac{k_B T \mu}{q} \tag{S9}$$

where $\mu = \frac{v}{E}$ is ions' mobility. Equation S9 is the well-known Nernst-Einstein equation which relates ions' mobility and diffusivity. From the above derivation, we deduce that Nernst-Einstein is exact no matter in bulk solution or in nanochannel when v is not very high (as the Stokes equation requires). Fig. 4b and Supplementary Figs. 22-23 show that D linearly correlates with $\frac{v}{qE}$ under various electrical field, E = 0.1 or 0.3 V·nm^{-1}. The slopes of $D \sim \frac{v}{qE}$ linear relation, 0.0239 J·C^{-1} (E = 0.1 V·nm^{-1}) and 0.0237 J/C (E = 0.3 V·nm^{-1}), are both consistent with the theoretical value (see eq. S9), $k_B T/e$ = 0.0257 J·C^{-1} (e = 1.602×10^{-19} C is elementary charge). These results further validate the Nernst-Einstein equation for ions' electromigration in nanochannel, and thus the mechanism revealed in this study for regulating D also holds true for regulating μ, since ions' D and μ just differ by a constant coefficient (equation S9).

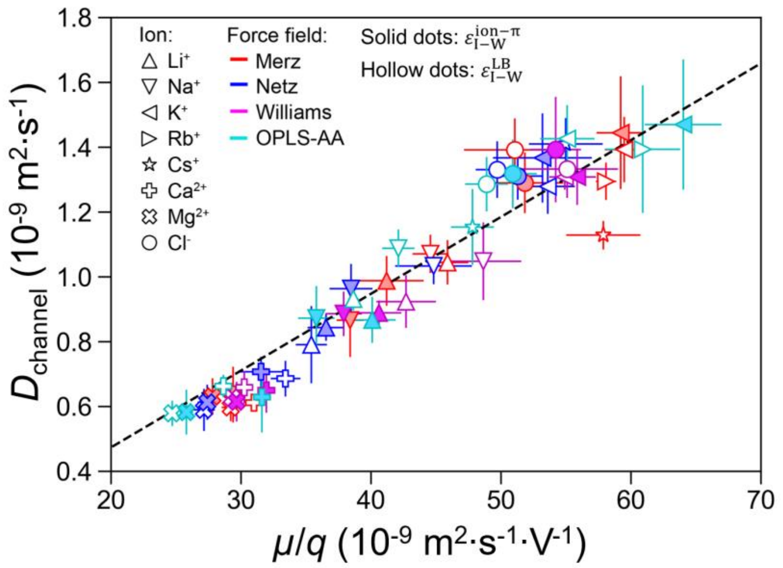

Supplementary Fig. 22 | The linear relation between ion's D and $\frac{v}{qE}$ under electric field of 0.3 V/nm in graphene 2D nanochannel. The black line is the linear fitting result (y = 0.0237·x, R^2 = 0.9423), whose slope is consistent with the theoretical value of 0.0257 J/C in the Nernst–Einstein relation. Error bars represent the standard error (n = 3 independent MD simulations), and the centers of error bars indicate the means. Source data are provided as a Source Data file.

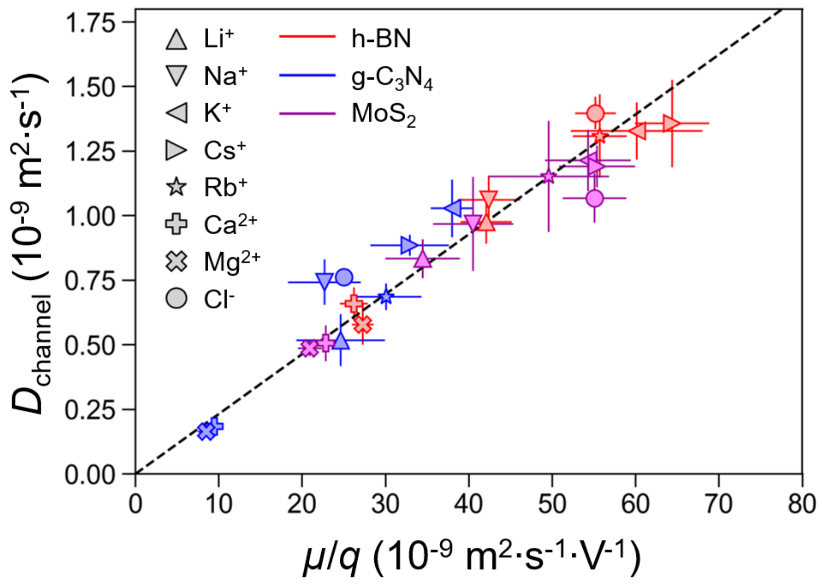

Supplementary Fig. 23 | The linear relation between ion's *D* and $\frac{v}{qE}$ under electric field of 0.1 V/nm for ions in 2D nanochannel constructed by h-BN, g-C_3N_4 or MoS_2, simulated with Merz FF. The black line is the linear fitting result (y = 0.0232·x, R^2 = 0.9388), whose slope is consistent with the theoretical value of 0.0257 J/C in the Nernst–Einstein relation. Error bars represent the standard error (*n* = 3 independent MD simulations), and the centers of error bars indicate the means. Source data are provided as a Source Data file.

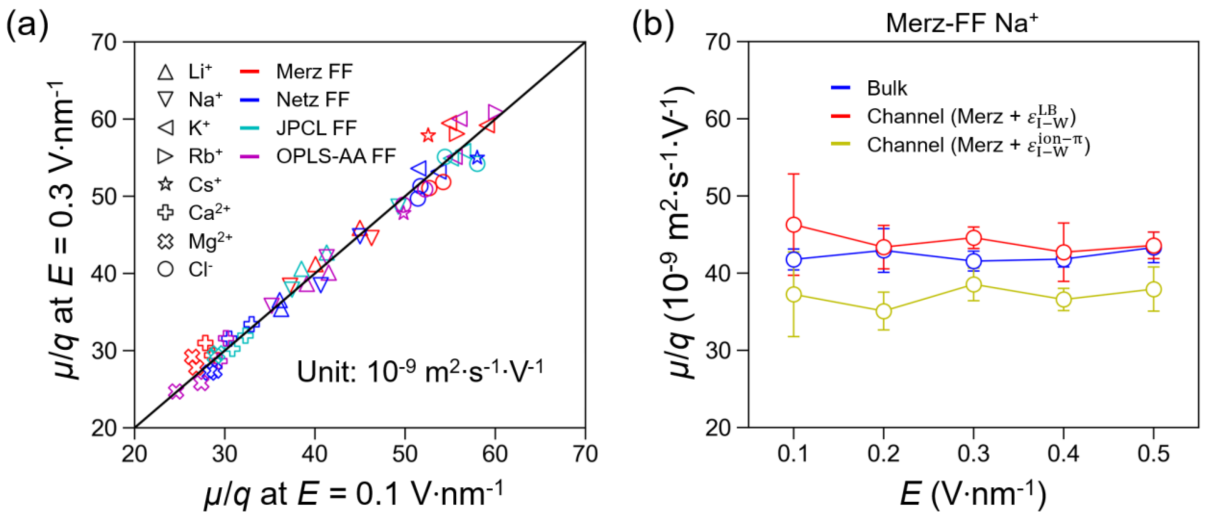

Supplementary Fig. 24 | The ion mobilities are constant for different electric fields. (a) In graphene 2D nanochannel, mobilities of all studied ions at $E = 0.1$ V·nm^{-1} are consistent with those at 0.3 V·nm^{-1}. (b) Mobility of Merz-FF Na^+ keeps constant under $E = 0.1 \sim 0.5$ V·nm^{-1}. Note the mobilities for Na^+ with $\varepsilon_{\mathrm{I-W}}^{\mathrm{ion-\pi}}$ are lower than those with $\varepsilon_{\mathrm{I-W}}^{\mathrm{LB}}$ in the nanochannel, as $d_{\text{ion-wall}}$ for Na^+ with $\varepsilon_{\mathrm{I-W}}^{\mathrm{ion-\pi}}$ decreases (Figs. 1-3 in the main text and relevant discussions). Error bars represent the standard error ($n = 3$ independent MD simulations), and the centers of error bars indicate the means. Source data are provided as a Source Data file.

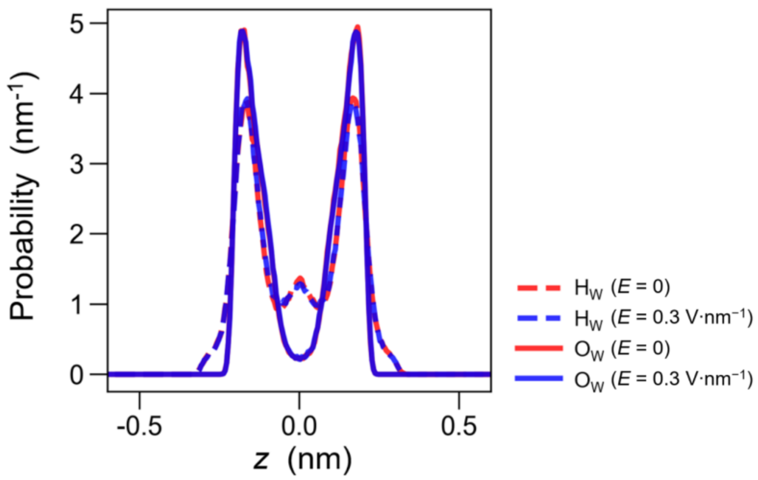

Supplementary Fig. 25 | Distribution profiles of water's oxygen (O_w) and hydrogen (H_w) atoms in graphene 2D nanochannel. The electric field of 0.3 V·nm^{-1} has a negligible effect on distribution profiles of O_w and H_w, indicating that the electric field hardly changes the structure of water layers. Source data are provided as a Source Data file.

Supplementary Table 8 | Simulation systems and resulting principles for ion transport in 2D nanochannels or in bulk solution.

Channel wall material	Ion	Ion concentration	Electric field (V/nm)	Constraint on ion	Principles
Graphene	Li^+, Na^+, K^+, Rb^+, Cs^+, Ca^{2+}, Mg^{2+}, Cl^-	Infinite dilution: 1 ion + 553 water molecules [a]	0	No constraint	$D_{channel}/D_{bulk}$ ~ $d_{ion\text{-}wall}$ correlation (Fig. 1b-1d, red parts in Fig. 4a)
				harmonic potential	$D_{channel}/D_{bulk}$ ~ $d_{ion\text{-}wall}$ correlation (Supplementary Fig. 8)
				Ion location fixed [c]	Quantitative mechanisms of "$\rho_{HS} \rightarrow \Delta F \rightarrow \tau \rightarrow I_{FACF}$" (Figs. 2&3, blue parts in Fig. 4a)<br><br>FACF method yields $D_{channel}/D_{bulk}$ (or D) values consistent with those yielded by MSD method. (Supplementary Figs. 13&14)
			0.1 ~ 0.5	No constraint	Nernst–Einstein relation holds true for ions in 2D nanochannels (Fig. 4b, Supplementary Figs. 22)<br><br>$\mu_{channel}/\mu_{bulk}$ ~ $d_{ion\text{-}wall}$ correlation (Fig. 4c, red parts in Fig. 4a)<br><br>Ion mobility keeps constant as electrical field increases from 0.1 to 0.5 V/nm. (Supplementary Fig. 24)
		0.1 M: 4 Cations + 4 Cl^- + 2212 water molecules [a]	0.3	No constraint	MD simulations reproduce the experimental ion mobilities of studied ions in graphene 2D nanochannel. (Supplementary Fig. 27)
hBN<br>MoS_2<br>g-C_3N_4	Li^+, Na^+, K^+, Rb^+, Cs^+, Ca^{2+}, Mg^{2+}, Cl^-	Infinite dilution: 1 ion + 587 (hBN), 703 (MoS_2) or 777 (g-C_3N_4) water molecules [a]	0	No constraint	$D_{channel}/D_{bulk}$ ~ $d_{ion\text{-}wall}$ correlation (Fig. 1e)
			0.1		Nernst–Einstein relation holds true for ions in 2D nanochannels (Fig. 4b, Supplementary Figs. 23)
bulk solution	Li^+, Na^+, K^+, Rb^+, Cs^+, Ca^{2+}, Mg^{2+}, Cl^-	Infinite dilution: 1 ion + 553 water molecules [b]	0	No constraint	MD simulations reproduce the experimental diffusivities of studied ions in bulk solution (Supplementary Fig. 26)
				Ion location fixed [c]	FACF method yields D_{bulk} values consistent with those yielded by MSD method. (Supplementary Figs. 13&14)
		0.1 M: 4 Cations + 4 Cl^- + 2212 water molecules [b]	0.1	No constraint	MD simulations reproduce the experimental mobilities of studied ions in bulk solution. (Supplementary Fig. 27)

[a]: The number of water molecules in nanochannels were determined based on the equilibrium water density in a water-soaked nanochannel under the pressure of 1 bar, as shown in our previous works[14]. The x-y size of these 2D nanochannels are 4.920×5.124 nm^2 (graphene), 5.260×5.206 nm^2 (hBN), 5.525×5.742 nm^2 (MoS_2)

or 5.705×6.176 nm^2 (g-C_3N_4). In the simulations of 0.1 M ion concentration, we doubled the x-y size of the graphene nanochannels to accommodate 4 cations and 4 Cl^- ions.

[b]: The number of water molecules corresponded to the bulk water density of 0.998 g/cm^3. In the simulations of 0.1 M ion concentration, the size of simulation system was expanded to accommodate 4 cations and 4 Cl^- ions (the water density keeps as the same).

[c]: Ions in nanochannel were fixed either at middle or edge position (see main text for explanations) to calculate ρ_{HS}, ΔF, τ and I_{FACF} (see Methods section calculation details)

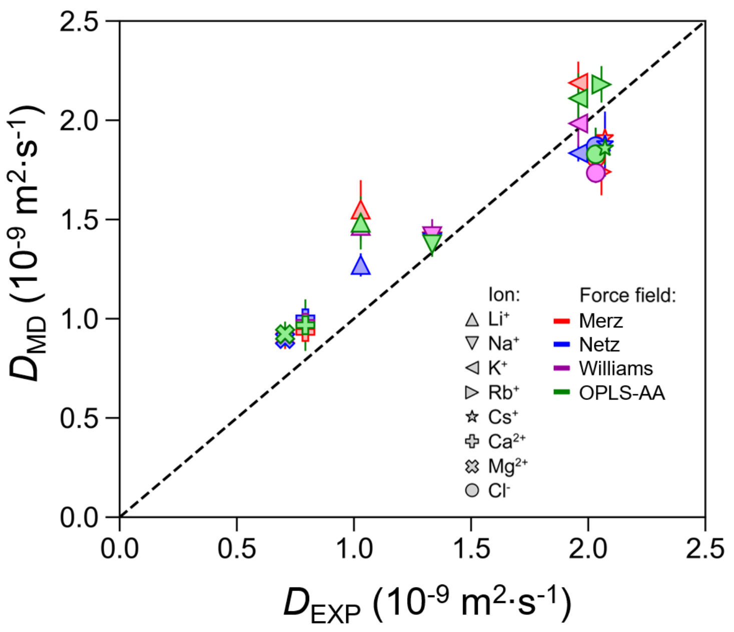

Supplementary Fig. 26 | Diffusivities of ions in bulk solution yielded by MD simulation (D_{MD}) are consistent with the experimental results[17] (D_{EXP}). To eliminate the system-size effect[18], D_{MD} in this figure were corrected as $D_{MD} = D^*_{MD} + \frac{2.837297 k_B T}{6\pi\eta L}$, where D^*_{MD} is the simulated diffusivity listed in Supplementary Table 3, k_B is Boltzmann's constant, T is the temperature, η is water's viscosity, L is the length of simulation box. Error bars represent the standard error (n = 3 independent MD simulations), and the centers of error bars indicate the means. Source data are provided as a Source Data file.

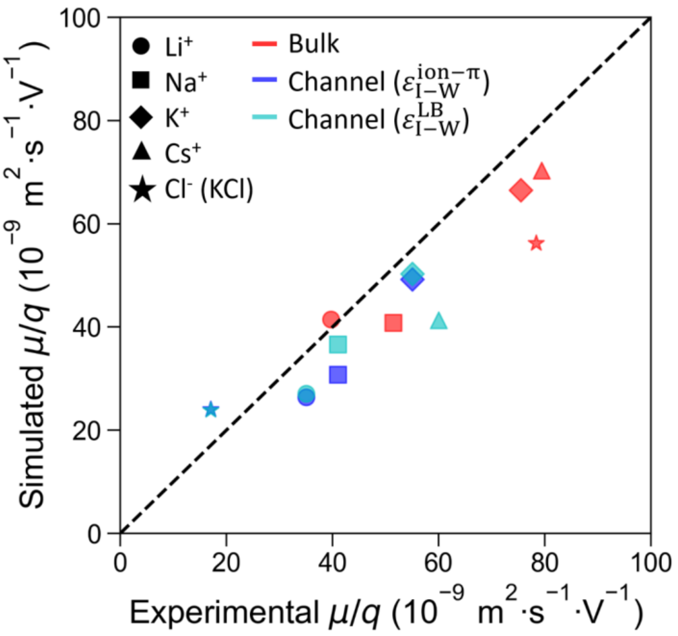

Supplementary Fig. 27 | Mobilities of ions yielded by MD simulation are consistent with experimental results[19]. MD simulations were performed with Merz FF (ε_{I-W}^{LB} indicates ε_{I-W} derived from LB mixing rule, while $\varepsilon_{I-W}^{ion-\pi}$ indicates optimized ε_{I-W}, see Supplementary Table 2) and salt concentration of 0.1 M in graphene nanochannel or in bulk solution. Source data are provided as a Source Data file.

Supplementary References

1. Li, P. F., Roberts, B. P., Chakravorty, D. K. & Merz, K. M. Rational Design of Particle Mesh Ewald Compatible Lennard-Jones Parameters for +2 Metal Cations in Explicit Solvent. *J. Chem. Theory Comput.* **9**, 2733-2748 (2013)
2. Li, P. F., Song, L. F. & Merz, K. M. Systematic Parameterization of Monovalent Ions Employing the Nonbonded Model. *J. Chem. Theory Comput.* **11**, 1645-1657 (2015)
3. Horinek, D., Mamatkulov, S. I. & Netz, R. R. Rational design of ion force fields based on thermodynamic solvation properties. *J. Chem. Phys.* **130**, 124507 (2009)
4. Mamatkulov, S., Fyta, M. & Netz, R. R. Force fields for divalent cations based on single-ion and ion-pair properties. *J. Chem. Phys.* **138**, 024505 (2013)
5. Williams, C. D., Dix, J., Troisi, A. & Carbone, P. Effective Polarization in Pairwise Potentials at the Graphene-Electrolyte Interface. *J. Phys. Chem. Lett.* **8**, 703-708 (2017)
6. Jorgensen, W. L., Maxwell, D. S. & Tirado-Rives, J. Development and testing of the OPLS all-atom force field on conformational energetics and properties of organic liquids. *J. Am. Chem. Soc.* **118**, 11225-11236 (1996)
7. Berendsen, H. J. C., Grigera, J. R. & Straatsma, T. P. The missing term in effective pair potentials. *J. Phys. Chem.* **91**, 6269-6271 (1987)
8. Werder, T., Walther, J. H., Jaffe, R. L., Halicioglu, T. & Koumoutsakos, P. On the water-carbon interaction for use in molecular dynamics simulations of graphite and carbon nanotubes. *J. Phys. Chem. B* **107**, 1345-1352 (2003)
9. Rajan, A. G., Strano, M. S. & Blankschtein, D. Ab Initio Molecular Dynamics and Lattice Dynamics-Based Force Field for Modeling Hexagonal Boron Nitride in Mechanical and Interfacial Applications. *J. Phys. Chem. Lett.* **9**, 1584-1591 (2018)
10. Chen, H. *et al.* Protein Translocation through a MoS2 Nanopore: A Molecular Dynamics Study. *J. Phys. Chem. C* **122**, 2070-2080 (2018)
11. Wang, Y. *et al.* Water Transport with Ultralow Friction through Partially Exfoliated g-C3N4 Nanosheet Membranes with Self-Supporting Spacers. *Angew. Chem. Int. Ed.* **56**, 8974-8980 (2017)
12. Liao, S. *et al.* Molecular Dynamics Simulation of Ion Adsorption at Water/Graphene Interface：Force Field Parameter Optimization and Adsorption Mechanism. *Chem. J. Chinese Universities* **44**, 184-195 (2023)
13. Tansel, B. Significance of thermodynamic and physical characteristics on permeation of ions during membrane separation: Hydrated radius, hydration free energy and viscous effects. *Sep. Purif. Technol.* **86**, 119-126 (2012)
14. Liao, S., Ke, Q., Wei, Y. & Li, L. Water's motions in x-y and z directions of 2D nanochannels: entirely different but tightly coupled. *Nano Res.* **16**, 6298-6307 (2023)
15. Daldrop, J. O., Kowalik, B. G. & Netz, R. R. External Potential Modifies Friction of Molecular Solutes in Water. *Phys. Rev. X* **7**, 041065 (2017)
16. Kowalik, B. *et al.* Memory-kernel extraction for different molecular solutes in solvents of varying viscosity in confinement. *Phys. Rev. E* **100**, 012126 (2019)
17. Haynes, W. M. *Handbook of Chemistry and Physics*. 99th edn, (CRC Press, 2018).
18. Yeh, I. C. & Hummer, G. System-size dependence of diffusion coefficients and viscosities from molecular dynamics simulations with periodic boundary conditions. *J. Phys. Chem. B* **108**, 15873-15879 (2004)

19. Goutham, S. *et al.* Beyond steric selectivity of ions using angstrom-scale capillaries. *Nat. Nanotechnol.* **18**, 596-601 (2023)
